# Toward Universal Forward Genetics: Using a Draft Genome Sequence of the Nematode *Oscheius tipulae* To Identify Mutations Affecting Vulva Development

**DOI:** 10.1534/genetics.117.203521

**Published:** 2017-06-19

**Authors:** Fabrice Besnard, Georgios Koutsovoulos, Sana Dieudonné, Mark Blaxter, Marie-Anne Félix

**Affiliations:** * École Normale Supérieure, Centre National de la Recherche Scientifique, Institut National de la Santé et de la Recherche Médicale, Institut de Biologie de l’École Normale Supérieure, Paris Sciences et Lettres Research University, 75005, France; † Institute of Evolutionary Biology, University of Edinburgh, EH8 9YL, United Kingdom

**Keywords:** *Oscheius tipulae*, genome assembly, mapping-by-sequencing, vulva development, *mig-13*

## Abstract

Mapping-by-sequencing has become a standard method to map and identify phenotype-causing mutations in model species. Here, we show that a fragmented draft assembly is sufficient to perform mapping-by-sequencing in nonmodel species. We generated a draft assembly and annotation of the genome of the free-living nematode *Oscheius tipulae*, a distant relative of the model *Caenorhabditis elegans*. We used this draft to identify the likely causative mutations at the *O. tipulae cov*-3 locus, which affect vulval development. The *cov-3* locus encodes the *O. tipulae* ortholog of *C. elegans mig-13*, and we further show that *Cel-mig-13* mutants also have an unsuspected vulval-development phenotype. In a virtuous circle, we were able to use the linkage information collected during mutant mapping to improve the genome assembly. These results showcase the promise of genome-enabled forward genetics in nonmodel species.

## Abstract (precis)

Understanding evolution requires the comparison of more than a few model species, and exploration of the genotype/phenotype relationship is limited...

A few model organisms have greatly contributed to biological research in the last decades, among them the nematode *Caenorhabditis elegans*. However, to tell conserved from specific features and understand the evolutionary process that gave rise to extant diversity, comparisons between different species at key phylogenetic positions are necessary. Genome sequences have been instrumental in model organism research, and the ongoing revolution in new genome sequencing and assembly technologies eases the once-daunting task of building such resources for any species. Draft assemblies can now be achieved within a few months at a reasonable cost, even by individual teams. Once a reference genome and gene annotation are available, other high-throughput sequencing techniques, such as RNA sequencing, can be used to explore genotype–phenotype interactions (Liu *et al.* 2015; Roux *et al.* 2015). Classical forward genetic approaches, *i.e.*, phenotype-based mutagenesis screens, are easy to perform and universal, provided the species can be cultured and crossed. Forward genetics has the huge advantage of identifying genes without prior knowledge or bias, which is particularly important in evolutionary comparisons. Such forward screens have been applied to many nonmodel species, but a remaining challenge is the identification of causative mutations and thus the function of target genes at the molecular level.

Massively parallel sequencing permits rapid identification of phenotype-causing mutations through “mapping-by-sequencing.” Mapping-by-sequencing has become a standard forward genetic approach in most model organisms, including *Arabidopsis thaliana* (James *et al.* 2013), *Saccharomyces cerevisiae*, *Danio rerio*, *Drosophila melanogaster*, and *C. elegans* (Schneeberger 2014). Mapping-by-sequencing strategies are generally based on the sequencing of bulk-segregant populations (Michelmore *et al.* 1991). Starting with a cross between the mutant strain of interest to a phenotypically wild-type but genetically different outcrossing strain, F_2_ grand-progeny individuals showing the recessive mutant phenotype are selected, and their DNA is pooled and sequenced. The recessive causative allele is necessarily homozygous for all individuals in this bulk mutant sample. For any polymorphic position between the two backgrounds, allele frequencies can be measured from mapped sequencing reads. If unlinked to the selected mutation, polymorphisms that distinguish the two parental strains will be found in the mutant pool at equal frequencies. However, if these markers are genetically linked to the causative mutation, the proportion of the allele from the wild-type background will decrease close to the mutated locus and approach zero in its immediate vicinity. Plotting allele frequencies along a reference genome will thus define a region of low wild-type allele frequency, surrounding the location of the causative mutation. The physical size of this interval will decrease with the number of meiotic recombination events in the F_2_ population (and, if limiting, with the number of available polymorphic positions).

In theory, if a mutant strain only differs from a nonmutant strain by a single mutation, comparing whole-genome sequencing data should reveal this polymorphism with no need for mapping (Nordström *et al.* 2013). In practice, genetic mapping information is required because a mutagenized strain and its nonmutagenized reference will have many spurious fixed differences: nonphenotype-causing mutations due to mutagenesis, or *de novo* spontaneous mutations fixed by drift in each strain. Technical noise, such as sequencing or mapping errors, can also contribute to observed variation. After one or several crosses, most of these variations can be excluded because they fall outside the mapping interval. Hence, sequencing bulk-segregant F_2_ populations and mapping allele frequencies on a reference genome is key to identifying the phenotype-causing mutation. Recently, mapping-by-sequencing has been applied to crop plants like rice (Abe *et al.* 2012; Fekih *et al.* 2013; Takagi *et al.* 2013), maize (Liu *et al.* 2012; Li *et al.* 2013), or barley (Mascher *et al.* 2014; Pankin *et al.* 2014), in which the genome is incomplete but high-resolution physical and genetic maps exist for each chromosome. Whether mapping-by-sequencing can be efficient in organisms with a fragmented or incomplete reference genome and no genetic map has not been tested.

The phylum Nematoda, which includes the model organism *C. elegans*, is an ideal target for the development of new species as model organisms amenable to forward genetics, because many species combine easy genomics and easy genetics. First, the relatively small size of nematode genomes, ranging from 20 to 400 Mb, ensures reasonable costs and good quality for most genome projects (Kumar *et al.* 2012). Second, the powerful genetics of *C. elegans* comes from its short life cycle and its androdioecious mode of reproduction, with selfing XX hermaphrodites that mate with X0 males in a facultative and controllable manner. Other nematode species sharing these features have therefore been chosen to perform forward genetics: *C. briggsae* (Gupta *et al.* 2007), *Oscheius tipulae* (Félix 2006), and *Pristionchus pacificus* (Sommer 2006). With *C. elegans*, these three free-living bacteriovorous nematodes belong to the Rhabditinae (De Ley and Blaxter 2002) [also referred as clade V (Blaxter *et al.* 1998) or 9 (Holterman *et al.* 2006; van Megen *et al.* 2009); see Figure 1]. Besides its mode of reproduction and easy culture, *O. tipulae* has been chosen for several reasons: the two-step, anchor-cell induction of vulval-precursor-cell fates and its simple vulval cell lineage (Félix and Sternberg 1997), its easy isolation from various regions of the world (Baïlle *et al.* 2008), and its phylogenetic position compared to *C. elegans* as an outgroup to *Caenorhabditis* species but an ingroup to *P. pacificus* (Blaxter *et al.* 1998). High-quality genome assemblies have been generated for *C. briggsae* (Stein *et al.* 2003) and *P. pacificus* (Dieterich *et al.* 2008). Classical techniques have been employed to map and identify genes involved in different traits with particular emphasis on the convergent evolution of self-fertile hermaphroditism, reviewed in Ellis and Lin (2014), and vulva development (Seetharaman *et al.* 2010; Sharanya *et al.* 2012, 2015; Sommer 2012; Kienle and Sommer 2013). In *P. pacificus*, genetic analyses of the evolution of sex muscles (Photos *et al.* 2006), gonad development (Rudel *et al.* 2008), dauer formation (Ogawa *et al.* 2009, 2011), and buccal tooth dimorphism (Bento *et al.* 2010) have been published. Inspired by the versatile and robust pipelines of mapping-by-sequencing routinely used for *C. elegans* (Minevich *et al.* 2012), we generated a genome assembly for *O. tipulae* and here test mapping-by-sequencing in this species.

**Figure 1:**
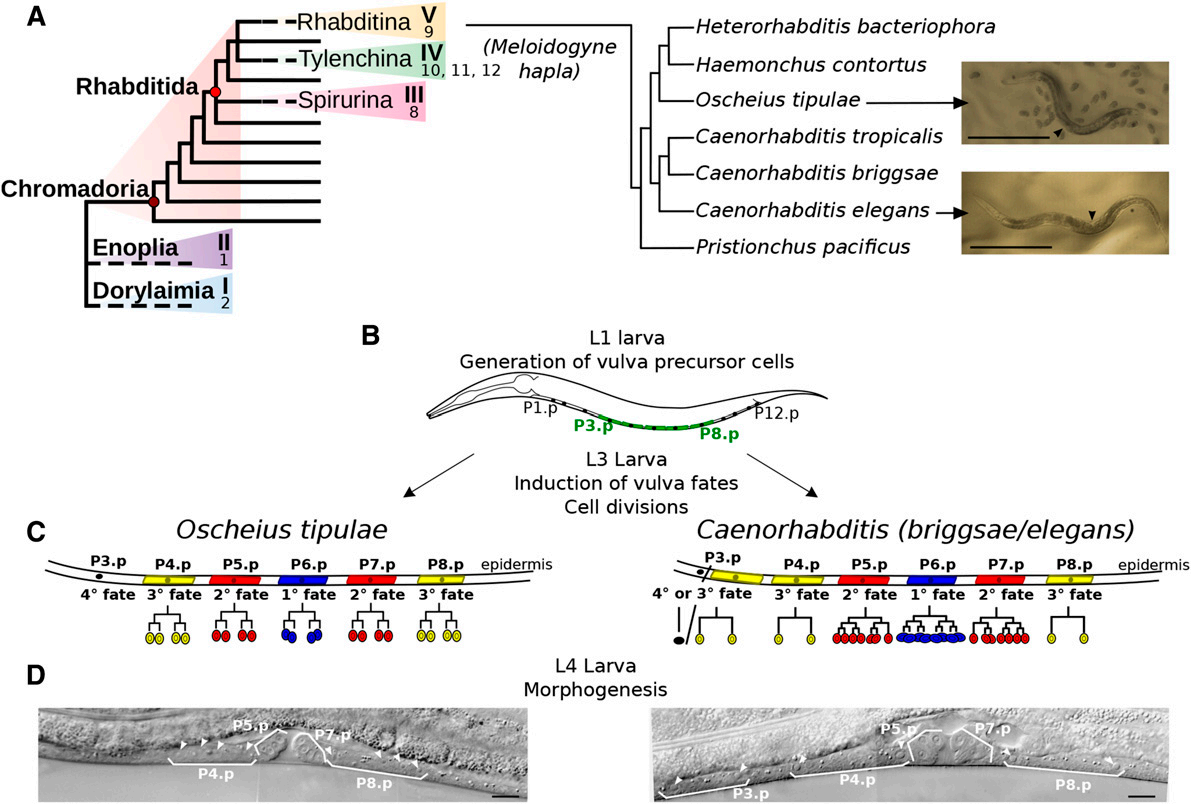
Phylogenetic relationships of *O. tipulae* and comparison of vulva development with *C. elegans*. (A) Cartoons showing (left) the systematic structure phylum Nematoda [clades I–V or 1–12 defined according to Blaxter *et al.* (1998), p. 199, and van Megen *et al.* (2009), respectively] and (right) the relationship of *O. tipulae* to key Rhabditina species discussed in the text. Pictures of young adult hermaphrodites of *O. tipulae* and *C. elegans* are shown on the right (arrowhead pointing to the vulva). Bar, 0.5 mm. (B–D) Vulva development in *O. tipuale* and *C. elegans*. (B) Ventral epidermal cells called P3.p to P8.p are specified as vulva precursor cells in young L1 larvae. (C) Conserved anteroposterior pattern of cell fates, despite variation in cell lineages: P6.p occupies the central position and adopts a 1° fate, P5.p and P7.p are induced to follow a 2° fate, and their daughters will form the border of the vulval invagination. Other cells (P3,4,8.p) differentiate into nonvulval epidermal fates, either with or without one round of division (3° or 4° fates, respectively). (D) Nomarski images of wild-type, L4-stage hermaphrodites (left, *O. tipulae*; right, *C. elegans*), highlighting the daughter cells of the Pn.p cells (arrowheads). Bar, 10 µm.

The available *O. tipulae* mutants have been obtained in forward genetic screens for mutations affecting vulva development and egg laying (Dichtel *et al.* 2001; Louvet-Vallée *et al.* 2003; Dichtel-Danjoy and Félix 2004). The nematode vulva connects the uterus to the outside and is required for egg laying and copulation. Vulva precursor cell specification is one of the best known developmental systems in *C. elegans* (Sternberg 2005), and the development of the vulva has become an important system for comparative studies in nematodes (Sommer and Bumbarger 2012). The *C. elegans* vulva develops from a group of six precursor cells aligned along the ventral midline of the animal, called P3.p to P8.p (Figure 1). While the vulva is under strong selective pressure for egg laying and mating (especially in obligate outcrossing species), a complex mosaic of change and stasis of different vulval developmental traits is observed among Rhabditinae (Kiontke *et al.* 2007). To date, most studies have focused on the conservation of the anteroposterior pattern of fates expressed by the three central precursor cells P5.p, P6.p, and P7.p across wide phylogenetic distances (Figure 1). Progeny of these three cells have specific division patterns and play specific roles in forming the mature vulva, with (usually) P6.p taking a central, primary (1°) fate, and P5.p and P7.p taking a secondary (2°) fate. Nematode vulval development is one of the best examples of how pervasive but cryptic evolution modifies the mechanisms of development despite an invariable output; a phenomenon known as “developmental system drift” (True and Haag 2001; Burgess 2011; Robinson 2011). Interestingly, the phenotypic spectrum of *O. tipulae* vulva mutants suggests substantial underlying evolutionary differences in specification and interaction compared to other species. In *O. tipulae*, all but 1 of the 34 vulval mutations so far isolated in forward genetic screens await molecular identification. Uncovering the molecular nature of the 33 mutants that are still uncharacterized in *O. tipulae* will help to unveil the mode and tempo of the vulva development system drift and possible innovations among Rhabditinae.

Here, we integrate a range of genomic tools to rapidly and cost effectively build a draft genome assembly and annotate genes in the nematode *O. tipulae*. Despite its fragmentation, we show that this draft assembly is a suitable platform for mapping-by-sequencing. As a proof of concept, we identify the vulva mutant gene *cov-3* (Louvet-Vallée *et al.* 2003) as the *O. tipulae* homolog of *C. elegans mig-13*. Finally, we show that linkage information collected during mapping of mutant alleles can be further used to detect mis-scaffolding and to group scaffolds into chromosome-scale linkage groups, improving the initial assembly, and providing useful information for further genetic mapping and mutant gene identification.

## Materials and Methods

### Nematode strains and culture

The *O. tipulae* reference strain CEW1, originally from Brazil, was used for forward mutant screens (Félix *et al.* 2001). The second wild strain that we used as a source of molecular polymorphisms is JU170, a strain sampled from soil in Sevilla, Spain, in 2000. This latter strain was chosen as it has a high genetic distance compared to CEW1, based on prior amplified fragment length polymorphisms (AFLP) analysis of a set of 63 wild *O. tipulae* isolates (Baïlle *et al.* 2008). *O. tipulae* mutant strains were generated as previously described (Félix *et al.* 2000; Dichtel *et al.* 2001). See Supplemental Material, Table S3, for the list of mutant alleles used in this study. These alleles were described in Dichtel *et al.* (2001), Louvet-Vallée *et al.* (2003), and Dichtel-Danjoy and Félix (2004), except for *mf33*, whose phenotype is only weakly penetrant and will be described elsewhere. *O. tipulae* strains were thawed from frozen stocks and cultured at 23° on NGM-plates seeded with *Escherichia coli* OP50, as previously described (Félix *et al.* 2000). The *C. elegans* strains used were Bristol strain N2 and the CF726 strain carrying the *mig-13*(*mu225*) *X* mutation, and they were cultured according to standard protocols (Brenner 1974) at 20°.

### Library preparation and next-generation sequencing

For genome assembly, genomic DNA was extracted from a mixed-stage growing population using reagents from the Puregene Core Kit A (QIAGEN, Valencia, CA). Data were generated from a 400-bp library and a 3-kb mate-pair or jumping library, following manufacturer’s instructions. The mate-pair library was constructed by the Centre for Genome Research, Liverpool. The 400-bp library was sequenced on the Illumina MiSeq platform (6.5 million 100-base read pairs and 20.7 million 300-bp read pairs). The paired-end library was sequenced on an Illumina HiSeq2500 (145.5 million 100-bp paired reads) (Table S1). Raw data have been submitted to the International Nucleotide Sequence Database Consortium (INSDC) under project accession no. PRJEB15512.

RNA was extracted from a mixed-stage growing population of *O. tipulae* CEW1, cultured in standard laboratory conditions. Poly(A)-enriched complementary DNA was prepared from the RNA by GATC (Konstanz, Germany) and normalized using reassociation kinetics. RNA sequencing was performed by GATC on the Roche GS FLX Titanium platform. A total of 592,650 reads (average length 369 bp) remained after filtering for quality. Raw transcriptome data are available in INSDC under project no. PRJEB15512).

For mapping-by-sequencing, genomic libraries and sequencing data from *O. tipulae* JU170 (INSDC project accession no. PRJEB19969) and mutant F_2_ pools (see project accessions in Table S3) used were generated by BGI. Short insert libraries (<800 bp) were paired-end sequenced on Illumina Hiseq2000, Hiseq2500, or Hiseq4000 with 100-bp reads to obtain 2.2 GB (∼40× coverage) of clean data per sample after manufacturer’s data filtering (removing adaptor sequences, contamination, and low-quality reads).

### Genome assembly

All software tools used (including versioning and command line main options) are summarized in Table S4. Raw reads were trimmed for adaptors using Cutadapt (Martin 2011) and low-quality bases, then corrected for sequencing errors based on k-mer content using Quake (Kelley *et al.* 2010) and JELLYFISH (Marçais and Kingsford 2011). Raw data were checked with FastQC (Andrews 2010) and a preliminary assembly generated with CLC Assembly Cell (CLC bio 2017) (Table S5). The CLC assembly was screened for contaminants using taxon-annotated, GC-coverage (TAGC) plots (Kumar *et al.* 2013). Only data deriving from *E. coli*, the food source, was identified as contaminant and the corresponding reads were removed. The optimal k-mer size for assembly of the cleaned read set was estimated using KmerGenie (Chikhi and Medvedev 2014). Nine different assemblers (Table 1) were used to generate preliminary assemblies and these were assessed using basic metrics, correctness of read alignment using ALE (Clark *et al.* 2013) and REAPR (Hunt *et al.* 2013), and biological completeness using Core Eukaryotic Genes Mapping Appoach (CEGMA) (Parra *et al.* 2007) and direct identification of ribosomal RNA genes and mitochondrial genome sequences. SPAdes (Bankevich *et al.* 2012) outperformed the other assembly tools in almost all aspects and was chosen as draft assembly nOt.1.0. An improved assembly limited to the nuclear genome (nOt.2.0) was generated by removing mitochondrial contigs and contigs of abnormally low coverage and by breaking all scaffolds where mis-assembly had been indicated from analysis of mapping plots and REAPR fragment coverage distribution (FCD) scores.

**Table 1:** Statistics of the assemblies generated by different assemblers

	MaSuRCA (Zimin *et al.* 2013)	SGA (Simpson and Durbin 2010)	Velvet (Zerbino and Birney 2008)	AbySS (Simpson *et al.* 2009)	CLC (CLC bio 2017)	Ray (Boisvert *et al.* 2010)	SOAPdenovo (Luo *et al.* 2012)	ALLPATHS-LG (Gnerre *et al.* 2011)	SPAdes (Bankevich *et al.* 2012)
Span (bp)	89,577,071	61,516,197	58,979,055	59,511,073	59,525,064	59,264,893	60,512,111	59,578,117	59,698,979
No. of scaffolds	6,646	5,619	786	782	520	147	313	151	203
Longest scaffold	134,185	836,405	1,580,233	1,581,252	2,304,523	3,256,501	2,993,798	3,772,824	4,597,891
N50	18,584	78,455	213,759	258,755	659,741	1,269,874	1,466,413	1,567,404	1,573,002
No. N’s	3,067,902	276,207	1,238	160,214	480,962	153,992	1,918,481	492,105	17,036
GC content (%)	44.5	44.5	44.5	44.5	44.5	44.5	44.5	44.5	44.5
Absolute REAPR score*^a^*	3.7	8.1	9.1	9.4	6.4	2.8	1.5	5.2	8.4
ALE score*^b^*	−2,909 × 10^6^	−934 × 10^6^	−482 × 10^6^	−467 × 10^6^	−976 × 10^6^	−1,110 × 10^6^	−982 × 10^6^	−978 × 10^6^	−291 × 10^6^

a REAPR absolute score measures the frequency of error-free bases and contigs, ranging from 0 to 1.

b ALE score is computed from the logarithm of the probability that the assembly is correct. ALE scores are negative: the closer to zero, the larger is the probability of the assembly of being correct.

### Gene prediction and orthogroup inference

Genes were predicted using a two-pass pipeline (Koutsovoulos *et al.* 2014) (see Figure S3) based on MAKER2 (Holt and Yandell 2011) and Augustus (Stanke *et al.* 2006), and using the transcriptome data as evidence. Repeats were identified in the assembly using RepeatModeler (Smit *et al.* 2013–2015). MAKER2 was run in an SGE cluster using the SNAP (Korf 2004) *ab initio* gene finder trained by CEGMA (Parra *et al.* 2007) output models, the GeneMark-ES *ab initio* finder, SwissProt proteins, and *O. tipulae* transcripts. The transcriptome data were filtered so that only reads >300 bases that had significant basic-local-alignment-search-tool (BLAST) similarity to *C. elegans* protein databases were kept. The MAKER2 predictions were used to train Augustus to generate a custom gene-finding profile for *O. tipulae*. Finally, Augustus was used with the gene-finder profile and *O. tipulae* transcripts to predict the final gene set. Not enough transcript evidence was available to train a model of untranslated regions (UTRs), and therefore no UTRs were annotated. Protein sets for *C. elegans* (*C*. *elegans* Sequencing Consortium 1998), *Dictyocaulus viviparus* (Koutsovoulos *et al.* 2014), *Meloidogyne hapla* (Opperman *et al.* 2008), and *P. pacificus* (Dieterich *et al.* 2008), downloaded from WormBase (http://www.wormbase.org/), were clustered with Orthofinder (Emms and Kelly 2015) using an inflation value of three.

### Synteny

*O. tipulae s*caffolds containing >100 predicted protein-coding genes were selected to perform synteny analyses between *O. tipulae* and *C. elegans*. Predicted proteins from these 36 scaffolds were compared to the *C. elegans* protein data set with BLAST to identify orthologous pairs. For each pair, the chromosome location of the *C. elegans* ortholog was identified. Hierarchical clustering was performed to group the scaffolds into groups based on the proportions of *C. elegans* chromosomal attributions (Figure S9).

### Mapping crosses

JU170 males were crossed with young mutant hermaphrodites of the desired recessive mutant genotype (in the CEW1 background) and several F_1_ cross-progeny were singled. In the F_2_ progeny, mutant animals were isolated based on the observation of the mutant phenotype (checked under Nomarski microscopy if necessary) and singled onto individual plates. These lines were amplified by selfing and allowed to grow until the *E. coli* food was just exhausted. For *cov-3* F_2_ mutant pool sequencing, independent mutant F_2_-derived populations (21 for *mf35* and 51 for *sy463*) were washed several times in M9 buffer (Stiernagle 2006) and stored at −80° as pellets. A similar mass of nematodes from each F_2_ line was then pooled for DNA extraction. For the F_2_ sequencing of other mutations, each F_2_-derived population was checked for the presence of the mutation in the homozygous state and allowed to grow until the *E. coli* food was just exhausted. Nematodes were directly pooled from different plates and washed in M9 buffer. DNA was then extracted using the Puregene Core Kit A (QIAGEN).

### Variant analysis, gene mapping, and identification

JU170 whole-genome sequencing data were analyzed to identify SNPs compared to the CEW1 reference genome. These variants were then used for genetic mapping of the mutants (listed in Table S3). Reads were mapped with bwa (Li and Durbin 2009) to the CEW1 assembly, mappings processed with the GATK tool suite (McKenna *et al.* 2010) version 3.3-0 and variants called with HaplotypeCaller using default parameters. We followed HaplotypeCaller’s authors’ recommendations of best practice (DePristo *et al.* 2011; Van der Auwera *et al.* 2013), realigning reads around indels and performing BQSR by bootstrapping a first call made with HaplotypeCaller. We analyzed the 300-bp CEW1 MiSeq data used for genome assembly with the same pipeline, after *E. coli* decontamination, as a control for variant calling. We then hard filtered a list of high-confidence SNPs of JU170 with conservative criteria, retaining homozygous positions covered by at least three reads in each strain, with a sequencing and mapping quality higher than 100 and 55, respectively, and a position noted as reference in CEW1 and variant in JU170. Sequencing data from pooled F_2_ mutants were analyzed with the same pipeline, except that variant calling was restricted to a list of JU170 SNPs previously established for faster computing (using the HaplotypeCaller option genotyping_mode GENOTYPE_GIVEN_ALLELES). Output VCF files were used to compute allele frequencies for each SNP on the JU170 list as the ratio of the number of reads with the JU170 allele over the total number of reads. These frequencies were plotted along each scaffold using custom R scripts. Scaffolds displaying a mean JU170 allele frequency <10% were selected as possibly linked to the candidate locus and retained for a second, unrestricted variant call. JU170 variants were filtered out from the output at this stage. We also systematically added for analysis the 47 scaffolds that do not carry SNPs between JU170 and CEW1 (0.1% of the genome). The functional impact of identified variants was assessed using snpEff (Cingolani *et al.* 2012) and used to prioritize candidate genes. Where two alleles of the same gene were analyzed, candidate gene lists were compared to exclude identical variations (likely initial background variations) and were inspected for independent hits to the same gene with a different noncomplementing mutation. When necessary, visual inspections of variations in aligned reads was performed with Tablet (Milne *et al.* 2013). Scripts used to automate this pipeline are available at: https://github.com/fabfabBesnard/Andalusian_Mapping.

### Sanger sequencing and gene validation

Four primers were designed to cover the *Oti-mig-13* coding region. *Oti-mig-13* fragments were amplified from strains homozygous for the four alleles of *cov-3* [*mf35*, *mf79*, *mf80*, and *sy463* (Louvet-Vallée *et al.* 2003)]. PCR products were verified on agarose gels, cleaned on columns, and sent for Sanger sequencing to Eurofins.

### X chromosome linkage of scaffolds and pyrosequencing

Linkage of scaffolds to the X chromosome was determined using directed pyrosequencing of F_1_ males from crosses (in both directions) between the CEW1 and JU170 strains. For each scaffold, we selected one polymorphic nucleotide position in the middle of the scaffold in an otherwise conserved context (no other variations in the 300 bp surrounding the variant) to ensure unbiased PCR. For each position, two PCR primers and one sequencing primer were designed (Table S6) using the pyrosequencer’s companion design software (PyroMark Q96 ID instrument from Biotage, Uppsala, Sweden). The primers were tested on parental strains. PCR using universal biotinylated primers and single-stranded PCR amplicon purification was performed as previously described (Duveau and Félix 2010). For each genotyping assay, in a successful mating plate, three individual adult F_1_ males and three individual adult F_1_ hermaphrodites were transferred individually into 10 µl worm lysis buffer containing proteinase K (200 mg/ml) and frozen at −20°. Nematodes were then thawed and lysed at 60° followed by 15 min at 95° to inactivate the proteinase. A total of 4 µl of worm lysate was used as PCR template. Pyrosequencing reactions were performed in the sequencing mode. High-quality DNA extracts from the parental strains were used as positive controls (separately and mixed to mimic a heterozygote) for each assay. Linkage to autosomes or the X chromosome was made if at least two male genotypes were concordant.

### Scaffold linkage analysis

For each mutant strain (see Table S3) and each scaffold, the mean frequency of alleles in the F_2_ mapping population whole-genome sequencing was extracted from the previous pipeline. All data sets included frequencies for the 144 scaffolds (over a total of 191 in nOt.2.0) that contained polymorphic positions between the strains CEW1 and JU170. Scaffolds and F_2_ mapping populations were clustered using the “heatmap.2” function of the “gplots” package in R.

### Microscopy

The vulval-cell phenotypes were determined during the early to mid-L4 larval stage using Nomarski microscopy.

### Data availability

All raw sequencing data supporting the conclusions of this article have been submitted to the INSDC. Sequencing data of genomic DNA and RNA from reference strain CEW1 (used for assembly) are available under project accession no. PRJEB15512. Whole-genome resequencing data of the mapping strain JU170 is under project accession no. PRJEB19969. Project accession numbers corresponding to the sequencing data of F_2_ mapping populations of the different strains used in this study are listed in Table S3. Annotations have also been submitted to the database European Molecular Biology Laboratory–European Bioinformatics Institute under the accession no. PRJEB15512.

All scripts used for mapping-by-sequencing and gene identification are available in https://github.com/fabfabBesnard/Andalusian_Mapping.

The genome assembly and annotation is available for browsing, exploration, and download at http://ensembl.caenorhabditis.org/index.html; and will be uploaded soon to WormBase (http://www.wormbase.org/).

## Results

### Assembly and annotation of the *O. tipulae* genome

We sequenced the genome of *O. tipulae* strain CEW1, which was used as the reference strain in all previous molecular and genetic studies (Blaxter *et al.* 1998; Félix *et al.* 2001; Ahn and Winter 2006; Félix 2006). Following the strategy suggested by the 959 Nematode Genomes project (Kumar *et al.* 2012), we generated data from two libraries (see *Materials and Methods* and Table S1). Paired-end 300-base Illumina MiSeq reads (∼50-fold genome coverage) were generated from a short insert library, and mate-pair 100-base Illumina HiSeq2500 reads (∼150-fold coverage) from a 3-kb virtual insert library. Before assembly, we cleaned the raw data by removing adaptor and low-quality bases, and performed error correction (see *Materials and Methods* and Figure S1). *E. coli* contaminating data were identified using TAGC plots and removed (Kumar *et al.* 2013) (Figure S2). We compared the performance of nine different assemblers (Table 1). All but one of the assemblers agreed on a genome size of ∼60 Mb, confirming evaluations based on k-mer counting. The assembly generated by SPAdes (Bankevich *et al.* 2012) had the highest quality metrics, with only 203 scaffolds >500 bp, N50 of >1.5 Mb, and only ∼17,000 undetermined bases. The SPAdes assembly also had the highest accuracy as assessed by ALE (Clark *et al.* 2013) and the third best by REAPR (Hunt *et al.* 2013), which are two reference-independent programs designed to track assembly errors (Table 1). From this first version, named nOt.1.0, we derived the nuclear genome assembly nOt.2.0 by removing three mitochondrial scaffolds and discarding 32 contigs which had very low coverage. We also broke 16 scaffolds that showed evidence for overscaffolding (see below). The final draft assembly spans 59,468,623 bases and includes 191 contigs >500 bp. Gene finding was performed as previously described (Koutsovoulos *et al.* 2014), using both *ab initio* predictions and evidence from RNA-sequencing data (Figure S3 and see *Materials and Methods*), and resulted in 14,938 gene predictions (Table 2). We assessed the completeness of the nOt.2.0 assembly using the CEGMA pipeline, and identified 97.6% complete and 99.2% partial genes out of the set of 248 conserved and likely essential genes (Parra *et al.* 2007). The *O. tipulae* genome is surprisingly small, with a span only 59% of that of the model species *C. elegans*. This reduction likely results from different factors (Table S2). Genes are less numerous (74% that of *C. elegans*) and have a denser packing (251 genes per Mb compared to 202 genes per Mb in *C. elegans*). They are also shorter overall (mean gene length is 81% of *C. elegans*) with shorter introns (160 bp *vs.* 339 bp), despite more introns per gene (8.5 *vs.* 6). The reduction in genome size is mirrored in the reduction in the span of intergenic DNA, and the repeat content of this intergenic DNA is much reduced. Overall repeat content in *O. tipulae* is 8.4% of the genome compared to 18.7% in *C. elegans*, mostly due to a much lower span of DNA elements (1.0% in *O. tipulae* compared to 9.9% in *C. elegans*). Comparing gene orthologies revealed no particular patterns of gene losses but indicated a reduced amount of species-specific genes, suggesting lower frequency of gene expansion and diversification in *O. tipulae* (Figure S4).

**Table 2:** Genomic characteristics of *O. tipulae*

	*O. tipulae* (assembly nOt.2.0)
Genome size (Mb)	59
Total intergenic span (Mb) (% of total genome)	21 (35.5%)
Total genic span (Mb) (% of total genome)	38 (64.5%)
Number of genes	14,938
Number of genes/Mbp	251
Mean/median transcript length (bp)	1,368/1,032
Mean/median exon length (bp)	160/132
Mean/median number of exons per gene	8.5/7
Mean intron length (bp)	160

### Mapping-by-sequencing identifies the *cov-3* locus

Our motivation to assemble the *O. tipulae* genome was to identify the molecular lesions affecting vulva development in a set of previously characterized mutant strains (Dichtel *et al.* 2001; Louvet-Vallée *et al.* 2003; Dichtel-Danjoy and Félix 2004). To establish this proof of concept, we chose the *cov-3* (*cov* standing for *competence and centering of vulva*) mutant. *cov3*-mutant *O. tipulae* display a partial loss of vulval competence with a highly penetrant anterior shift of the vulval fate pattern (Louvet-Vallée *et al.* 2003). This phenotype has only recently been described in *C. elegans*, occurring at low penetrance in some Wnt pathway mutants (Milloz *et al.* 2008; Grimbert *et al.* 2016); suggesting that the coupling of competence and centering differs between the two species. Four alleles of *cov-3* were available, permitting independent confirmation of candidate genes.

For genetic mapping, we chose the strain JU170. This wild *O. tipulae* isolate is genetically distant from CEW1 (the genetic background of all mutants) based on AFLP (Baïlle *et al.* 2008). We resequenced JU170 to ∼40-fold coverage and identified SNPs and indels that distinguish CEWI and JU170. We found one SNP every 95 bp on average, and a total of 632,027 SNPs (see Figure S5). For comparison, the genetically distant strain used routinely in *C. elegans* mapping-by-sequencing (CB4856) has an average SNP density of 1 SNP every 1000 bp (Hillier *et al.* 2008; Minevich *et al.* 2012), which is 10-fold less.

**Figure 2:**
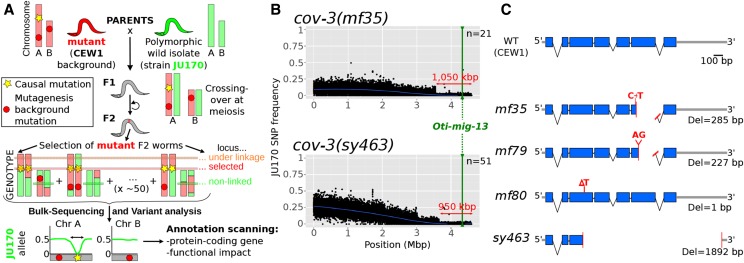
Mapping-by-sequencing of *O. tipulae* vulva mutants and identification of *cov-3* mutations in the *Oti-mig-13* gene. (A) Principle of the mapping-by-sequencing approach, involving the wild isolate JU170 as a mapping strain and whole-genome resequencing of a bulk of mutant F_2_ grand-progeny (see text for details). The phenotype-causing mutation is mapped genetically by the cross as the region of low frequency of JU170 alleles. Final identification requires scanning of this interval for variations specific to the mutant background. (B) JU170 allele frequency plots in scaffold 1 (genome version nOt.2.0.) in bulk-sequencing data generated with the independent *cov-3* alleles *mf35* and *sy463* (upper and lower plots respectively). On each plot, the blue line is a local regression of the JU170 allele frequency, the red arrow indicates the mapping interval size, the green line the position of the *mig-13* gene, and *n* the number of F_2_ lines pooled in each mapping population. (C) Cartoon depicting the structure of the wild-type *Oti-mig-13* gene and the alterations found in all independent alleles isolated so far. Mutations are indicated in red, exons are blue boxes, introns thin black lines, and intergenic regions thick gray lines. Del, deletion; WT, wild type.

We crossed *cov-3* hermaphrodites (alleles *mf35* and *sy465*) with JU170 males and selected F_2_ grand-progeny displaying the recessive *cov-3* phenotype. After amplification by selfing, F_2_-derived populations were pooled and genomic DNA extracted and sequenced (see *Materials and Methods* and Figure 2). To identify the *cov-3* mutations, a first variant call was performed only on known JU170 SNPs, and their frequencies were plotted genome wide. Scaffolds containing the lowest JU170 allele frequency were retained. A second-pass call scanned these scaffolds for all other variants (excluding JU170 and other background variations). This analysis retrieved only eight candidate mutations which were prioritized according to their functional impact. In the *sy463* pool, a putative 38-bp insertion was predicted to cause a frameshift in the *nOt.2.0.1.t01002* gene, homologous to *Cel-mig-13*. However, inspection of read pairing revealed a much larger deletion (1888 bp) in this gene. No SNP variants were called at this locus for the *mf35* pool, but inspection revealed a 283-bp deletion affecting the same gene (Figure S6). Such large deletions are expected to be less frequent than SNPs following EMS mutagenesis (Flibotte *et al.* 2010) and the variant toolkit we used is not optimal for identification of large indels.

Both mutations are predicted to result in truncation of the expressed protein, and thus are likely to be loss-of-function alleles. In the F_2_ bulk-segregant data, JU170 allele frequency displayed a clear drop in the ∼1 Mb at the end of scaffold nOt.2.0.scaf00001, an interval that contains ∼200 gene predictions including *Oti-mig-13* (Figure 2). We conclude that *Oti-mig-13* (*nOt.2.0.1.t01002*) is the best candidate gene for the *cov-3* locus. To confirm this, the *Oti-mig-13* locus was amplified and directly sequenced from all four available, independently isolated *cov-3* alleles: *sy463*, *mf35*, *mf79*, and *mf80*. The deletions predicted from whole-genome resequencing were confirmed in *sy463* and *mf35*. In the trimethylpsoralen-ultraviolet (TMP-UV)-induced alleles *mf79* and *mf80*, two new frameshift-causing deletions were found, and these are thus also likely to be loss-of-function alleles (Figure 2). We have thus been able to use a draft genome assembly to identify a locus by mapping-by-sequencing. The *O. tipulae cov-3* locus is orthologous to *C. elegans mig-13*. Following standard nematode genetics nomenclature procedures, we redesignate *Oti-cov-3* as *Oti-mig-13*.

### The role of *mig-13* in vulva development has changed between *C. elegans* and *O. tipulae*

**Figure 3:**
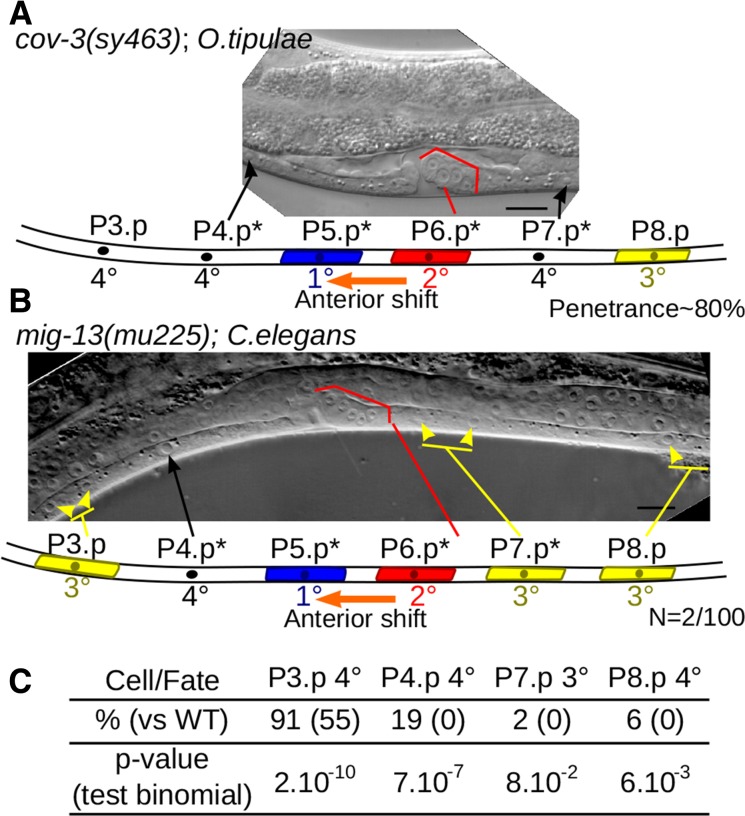
Conservation and evolution of MIG-13 between *C. elegans* and *O. tipulae*. (A) Typical phenotype of a *Oti-cov-3* mutant, shown in a Nomarski picture and an interpretation cartoon below: an anterior shift (1° fate shifted from P6.p to P5.p) is coupled to a reduced competence of vulva precursor cells. * indicates Pn.p cells with a modified fate compared to the wild type. (B) A *cov-3*-like phenotype can be observed at very low frequency in a *mig-13* null mutant, *Cel-mig13*(*mu225*), of *C. elegans*. (C) Reduced competence of P3,4,8.p cells in *Cel-mig-13*(*mu225*) is indicated by increased frequency of 4° fate *vs.* 3° fate. WT, wild type.

The MIG-13 protein is predicted to be a single-pass, *trans*-membrane protein that contains two protein–protein interaction domains (a CUB domain and an LDL-receptor repeat), both extracellular. *C. elegans* and *O. tipulae* MIG-13 proteins are quite similar (48% aa identity and 63.5% similarity), with higher identity in CUB and LDL functional domains (Figure S7). Similar organization is observed in MIG-13 homologs in other nematodes. In *C. elegans*, *mig-13* is necessary for the anterior migration of the neuroblasts of the QR lineage (Sym *et al.* 1999), but *Cel-mig-13* was not known to play any role in vulva development. While *O. tipulae cov-3* mutants display a partially penetrant egg laying-defective phenotype, the *C. elegans mig-13* mutants are not defective in egg laying. We further investigated the *Cel-mig-13*(*mu225*) null mutant using Nomarski microscopy on a large number of animals. We observed an anterior shift of the 1° fate on P5.p, associated with decreased P4.p competence, a phenotype identical to the *O. tipulae mig-13* phenotype, with very low (2%) penetrance. Penetrance of this phenotype is 80% in *O. tipulae* (Louvet-Vallée *et al.* 2003) (Figure 3, A and B). We also observed a more penetrant reduction in competence of vulval equivalence group cells in *Cel-mig13*(*mu255*), where P(3,4,8).p adopt the noncompetent fused 4° fate more frequently than the competent uninduced 3° fate (Figure 3C). Thus, *mig-13* does play a role in vulva development in *C. elegans*, but the impact of its loss of function is reduced compared to *O. tipulae*, especially as far as the centering defect is concerned. The difference in the mutational phenotypic spectrum between the two species is thus likely to be due to a quantitative rather than a qualitative evolution of the contributions of *mig-13* to the vulva genetic network. This example demonstrates the power of streamlined forward genetics in *O. tipulae*, as it both uncovers the evolution of developmental mechanisms hidden by a highly conserved cell-fate pattern, and also reveals new aspects of *C. elegans* development, even in a well-studied system such as vulva formation.

### Improvements of the *O. tipulae* genome assembly using genetic linkage data

**Figure 4:**
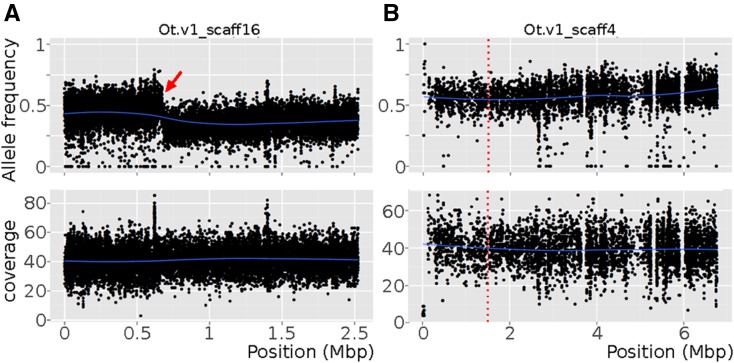
Mapping-by-sequencing detects mis-assembly. Variant analysis was performed using the preliminary assembly (nOt.1.0). Plots obtained from mapping-by-sequencing data set from the *cov-3*(*sy463*) mapping population. JU170 allele frequency is plotted in upper graphs for each SNP along (A) contig 16 or (B) contig 4 and the coverage is indicated below. Blue lines are local regressions of the allele frequency or of the coverage. (A) Contig 16: ← indicates a sudden shift in JU170 allele frequencies in the scaffold. (B) Contig 4: a REAPR FCD threshold score was deduced from plots with obvious breaks and applied to all scaffolds. Overscaffolding was detected in scaffold 4 (red vertical dotted line) in the absence of any conspicuous break in SNP frequency.

The JU170 allele frequency plots provide genome-wide information about genetic linkage that can be used to improve the genome assembly, both in identifying errors in assembly and in superscaffolding into linkage groups. Since many F_2_ lines are pooled in each data set, recombination events are averaged out and JU170 allele frequency (with mean of ∼0.5) should vary continuously, especially along scaffolds unlinked to the selected mutations. Following precedents (Leshchiner *et al.* 2012), we used abrupt breaks in JU170 allele frequency in the *mf35* and *sy463* data sets as indications of mis-assembly. We confirmed overscaffolding by direct inspection of the aligned reads in 11 cases (Figure 4A). From the REAPR FCD score for each broken scaffold, we estimated heuristically an FCD cutoff value, and broke five additional scaffolds that were not highlighted in the allele frequency plots (Figure 4B). Detection of mis-assemblies using allele-frequency plots is highly dependent on the position of the mapped mutant locus and the number of pooled F_2_ animals. However, allele frequency plots provide evidence-based criteria to inform cutoff parameters to assess the correctness of the whole assembly. The modifications outlined above were integrated in assembly version nOt.2.0.

**Figure 5:**
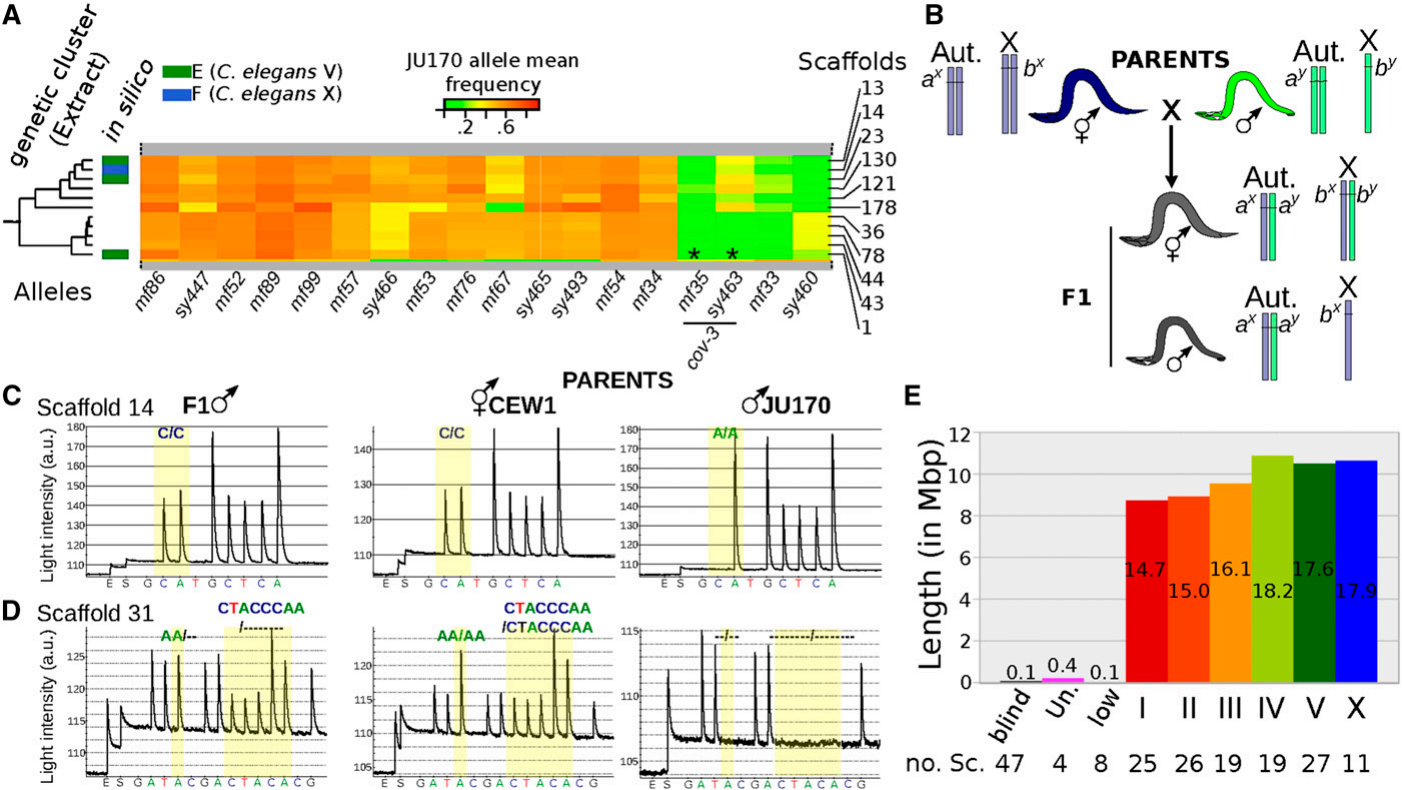
Building a chromosome-scale assembly of the *O. tipulae* draft assembly. (A) The *O. tipulae* cluster of scaffolds that correspond to the X chromosome. This cluster was selected from a global clustering based on JU170 allele mean frequency across 18 F_2_ mapping populations. The entire heatmap is shown in Figure S8. Each column of the heatmap corresponds to an independent F_2_ mapping population (Table S3) and each rectangle to the mean frequency of JU170 alleles in a scaffold. Scaffold identifiers are listed on the right side of the heatmap and the color scale is given above. If available, the prediction made from orthology-driven clusters (Figure S9) is indicated on the left side of the heat map. * indicates scaffolds where the causative *cov-3* mutation has been found. (B) Method to distinguish autosome-linked *vs.* X-linked loci. *a* and *b* loci are on an autosome (Aut.) and the X chromosome, respectively, and are polymorphic between two strains (alleles *x* and *y*). While F_1_ hermaphrodites are heterozygotes for both loci, F_1_ males are homozygotes for the X-linked locus and bear the maternal allele. (C and D) Pyrograms of an F_1_ male progeny (left) from a CEW1 hermaphrodite (middle) and a JU170 male (right): genotyping was performed with polymorphic markers in scaffold 14 and 31 (assembly nOt.2.0.), which are placed in genetic clusters (C and D), respectively. Scaffold 14 is linked to X while scaffold 31 is linked to an autosome. (E) Cumulative length of each cluster of scaffolds. Numbers within bars are percent of the whole assembly, numbers below the chart count the number of scaffolds in each cluster. ♀, female; ♂, male; blind, scaffolds bearing no genomic variants between JU170 and CEW1; low, scaffolds with consistent low mean JU170 allele frequency; roman numbers, chromosomes; Un., unplaced scaffolds.

A second kind of information present in JU170 allele frequencies is genetic linkage between scaffolds. In an F_2_ mapping population, a scaffold with a JU170 allele frequency significantly <0.5 indicates linkage to the mutation of interest. Scaffolds displaying a low mean JU170 allele frequency in the *sy463* data set were also consistently low in the *mf35* data set (see Figure 2B, Figure 5A, and Figure S8). We reasoned that we could infer linkage over the whole genome by analyzing similar data for several independent loci distributed over the genome. Following the same strategy as for *cov-3*, we generated and sequenced F_2_ mapping populations from a cross between JU170 and 16 additional strains from our *O. tipulae* mutant collection, corresponding to 14 different genetic loci (see Table S3). Using the same pipeline as for *cov-3*, the genes mutated in these strains were almost all identified and they will be described in a future article focusing on the evolutionary changes in vulva development between *O. tipuale* and *C. elegans*. Here, we only extracted the mean JU170 allele frequency of all scaffolds from each independent mapping data set, which does not require the causal mutations to be found. Using this approach, we sorted the genome into large chromosome-scale clusters, but which remain unordered (Figure S8).

While nematode genomes evolve rapidly, retention of chromosomal linkage in the absence of close synteny has been observed in several comparisons (Stein *et al.* 2003; Ghedin *et al.* 2007; Dieterich *et al.* 2008). We produced a *C. elegans* chromosome homology profile for each of the 36 largest scaffolds (>100 kb, 79% of the assembly span) (Figure S9). For each scaffold, we observed that a majority of the genes had orthologs located on a single *C. elegans* chromosome. This pattern was strongest for genes mapping to *C. elegans* chromosomes I, II, III, IV; and weaker for chromosomes V and X. Clustering of these orthology-based profiles generated six groups (labeled A–F in Figure S8 and Figure S9) that are likely to represent the six *O. tipulae* chromosomes observed by microscopy (Ahn and Winter 2006). We reasoned that if these orthology-driven clusters represent real chromosomes, they should also cluster by linkage. Indeed, orthology-driven clusters A, B, C, and D were fully preserved in the linkage clusters. We thus labeled by homology the corresponding clusters as part of *O. tipulae* chromosomes I, II, III, and IV, respectively, because the majority of genes in these linkage groups have their *C. elegans* ortholog in the corresponding chromosome (Figure S9).

In contrast, two scaffolds assigned *in silico* to group E and one assigned to group F were not genetically linked to other scaffolds of the respective group. Consideration of their content of orthologs suggested they should instead be swapped between these two chromosomes (Figure 5A and Figure S8). It was not possible to assign a clear *C. elegans* chromosome homology for these two remaining genetic clusters since they each have many orthologs in both *C. elegans* chromosomes V and X (Figure S9). To determine which *O. tipulae* chromosome is the sex chromosome, we directly genotyped F_1_ males from a cross between the reference strain CEW1 and the polymorphic strain JU170. Markers were designed from the larger scaffolds of groups E and F and animals were genotyped by pyrosequencing. X0-males will be hemizygous for any X-linked markers, but diploid and thus heterozygous for autosomal markers that distinguish CEWI and JU170 (Figure 5, B–D) (Srinivasan *et al.* 2002). This strategy identified group E as representing the X chromosome, confirming previous observations of X-linkage for *cov-3* (Louvet-Vallée *et al.* 2003) and *mf33* (M.-A. Félix, unpublished data). *Cel-mig-13* is on the *C. elegans* X chromosome. This assignment suggests that there have been substantial rearrangements involving what are now the *C. elegans* and *O. tipulae* V and X chromosomes. The genotyping also identified an additional mis-assembled scaffold (scaffold 8, Figure S10). We further annotated our preliminary chromosome assembly with the scaffold position of telomeres (File S1).

The scaffolds assigned to a chromosome corresponded to 99.5% of the genome. The remaining scaffolds were all small (Figure 5E). A group of 47 scaffolds that bear no genomic variants between JU170 and CEW1 cannot be mapped in this cross. Four scaffolds had a consistently high JU170 allele frequency across all genetic mapping data sets and are likely to be regions that happened to be genetically unlinked to all loci that were mapped in this study. Eight scaffolds had a consistently rather low mean JU170 allele frequency (20–25%) and clustered together. Their low frequency of JU170 alleles could be an artifact due to problems of read mapping in divergent, repeated, or mis-assembled regions, or may be due to transmission distortion in the cross between two wild isolates as seen in *C. elegans* (Seidel *et al.* 2008) or *C. briggsae* (Ross *et al.* 2011). For our mapping-by-sequencing approach, we flagged putative causative mutations associated with these scaffolds, but will return to them to attempt linkage attribution in the future.

## Discussion

### A draft assembly with a high quality provides a useful and versatile resource for a new nematode species

We assembled a rapid draft assembly for the reference CEWI strain of *O*. *tipulae* from relatively inexpensive Illumina short-read data. The assembly has good contiguity and gene content metrics, and appears to represent the *O. tipulae* genome well. It ranks among the better nematode genome assemblies alongside the complete *C. elegans* and *Onchocerca volvulus* assemblies and the almost complete *Strongyloides ratti*, *C. briggsae*, and *Candida tropicalis* assemblies, on which considerable finishing effort has been expended (*C*. *elegans* Sequencing Consortium 1998; Stein *et al.* 2003; Hillier *et al.* 2007; Koboldt *et al.* 2010; Ross *et al.* 2011; Cotton *et al.* 2016; Hunt *et al.* 2016). The high quality of the *O. tipulae* assembly is likely to be a result of the high homozygosity in the inbred strain sequenced (Barrière *et al.* 2009) and the small genome size (and concomitant reduced contribution of repeats, Table S2). The total genome length in our assembly is 40% shorter than a previous estimate from reassociation kinetics (Ahn and Winter 2006). We found no evidence for missing genetic content, or a large span of overcollapsed repeats. It is perhaps more likely that the reassociation kinetics-based estimate is in error, as was observed for *A. thaliana* [actual genome span of ∼135 Mb (The Arabidopsis Genome Initiative 2000), flow cytometry estimate of ∼150 Mb (Bennett *et al.* 2003), reassociation kinetics estimate of 70–80 Mb (Leutwiler *et al.* 1984)].

Regardless of the specific advantages of *O. tipulae*, achieving high-quality draft assemblies at reasonable cost is now feasible for a wide range of species. Advances in sequencing technologies and assembly algorithms can be combined to enable high contiguity assemblies, even from highly heterozygous organisms. For example, Fierst *et al.* (2015) recently produced a 131-Mb assembly of the highly polymorphic outcrossing *C. remanei* in only 1600 scaffolds, using a mix of short insert and mate-pair libraries, as we did here. We also found marked differences between assemblers, even those based on the same underlying algorithm. Comparisons of assembly toolkits have been made in several “Assemblathon” competitions, and from these it is clear that, while some assemblers do perform better consistently, customization of approach is key to optimal assembly (Earl *et al.* 2011; Bradnam *et al.* 2013). As the assumptions made in the coding of different assemblers may interact differently with the particular patterns of genome structure and diversity present in a target species, it may still be useful to assess several different assemblers in parallel for each new genome.

The *O. tipulae* assembly could still be improved. Long-read sequence data from Pacific Biosciences, Oxford Nanopore, or 10× Genomics platforms could be used to further contiguate the genome. Long-range physical mapping using the BioNano or OpGen optical-mapping platforms could be used to superscaffold the existing assembly. Both of these approaches could yield chromosomal-sized scaffolds. Traditional genetic map production from a large mapping cross could also be used to bin and order scaffolds in a linkage map, and validate the sequence- or physical-based assembly. Genotyping-by-sequencing approaches such as restriction site-associated DNA sequencing (Baird *et al.* 2008; Baxter *et al.* 2011; Fierst *et al.* 2015) or other reduced-representation sequencing methods would provide the density of markers required at minimal cost. Low-coverage, whole-genome skimming; for example, of recombinant inbred lines as applied in *C. elegans* (Li *et al.* 2006; Doroszuk *et al.* 2009; Rockman and Kruglyak 2009), *C. briggsae* (Hillier *et al.* 2007; Ross *et al.* 2011), and *P. pacificus* (Srinivasan *et al.* 2002); would serve the same goal. We were able to leverage the data generated from bulk-segregant identification of selected markers in a “virtuous circle” to also improve the assembly, binning scaffolds representing 99.5% of the assembly into putative chromosomal groups and will continue to do so with further mutations.

A complete assembly of the *O. tipulae* genome will require significant additional effort, but until that goal is achieved, we have shown that the existing assembly is a sufficient substrate for mapping of mutants and identification of genes. It is also highly informative concerning genome evolutionary dynamics in the Rhabditinae. The genome is available for browsing and download at http://ensembl.caenorhabditis.org/Oscheius_tipulae_not2/Info/Index.

### Toward universal forward genetics?

For the first time, we successfully identified without a candidate-gene approach a phenotype-causing gene in *O. tipulae*, namely the *Oti-mig-13* gene corresponding to the previously described *cov-3* vulva mutants (Louvet-Vallée *et al.* 2003). Our pipeline is similar to those developed for model species (Schneeberger *et al.* 2009; Minevich *et al.* 2012; James *et al.* 2013). The fragmentation of the genome into ∼200 contigs did not impair our ability to identify causative lesions. Three of the four mutations we identified in *Oti-mig*-13 were large deletions, including two derived from EMS mutagenesis screens. While TMP-UV mutagenesis is chosen because of its propensity to induce deletions, mutations induced by EMS are thought to mainly comprise G/C to A/T transitions (Anderson 1995); but they can also include a significant proportion of deletions, especially after screens for strong loss-of-function mutations (Flibotte *et al.* 2010; *C*. *elegans* Deletion Mutant Consortium 2012).

Our results suggest that most good-quality draft genome assemblies will be sufficient to allow the identification of loci identified by forward genetics. Methods have been developed that directly identify fixed differences in raw whole-genome sequencing data without mapping to an assembly (Nordström *et al.* 2013). This linkage- and reference-free strategy is particularly useful in organisms, such as plants, with large and repetitive genomes, because assembling such genomes is still challenging (Schneeberger 2014). However, this approach tends to output more candidate variants, and cannot exclude candidates by linkage. This is critical, as downstream validation of candidates is time consuming and occasionally not technically possible in nonmodel species. For Nematoda, where genomes are generally small, a pipeline with a *de novo* reference assembly and mapping by bulk-segregant analysis is very efficient.

Many nematode species are attractive subjects for laboratory research, thanks to their small size, fast life cycle, large broods, and simplicity of culture. The fact that many species can be cryopreserved simplifies genetic approaches. Many genetically diverse isolates are available from wild sampling collections for each species for generation of mapping populations. Facultative selfing has evolved multiple independent times in the phylum, and this, as in *C. elegans*, significantly simplifies genetic analyses. The homozygosing effect of selfing also ensures easier genome assembly, but high-quality genomic resources can still be built for obligate outcrossers, such as *C. remanei* (Fierst *et al.* 2015). Thus, many new nematode species, selfing or outcrossing, could now be turned into genetic model systems. The real bottleneck will be the development of functional genetic tools to validate the identified candidate genes. However, the revolution brought by the CRISPR-Cas9 system for genome editing may solve this issue, as shown by the recent successful implementations of this versatile technique in various nematode species other than *C. elegans*, including *P. pacificus* (Witte *et al.* 2015, p. 201) and *C. briggsae* (Culp *et al.* 2015).

### Evolution of development of the vulva in nematodes

We exemplify the relevance of the comparative genetic approach with our analyses of the evolution of the role of *mig-13* in nematode vulva development. First, this gene would not have been investigated in a targeted reverse genetic study: phenotype-based screens ensure unexpected findings about genetic innovation. Second, although *C. elegans* vulva development has been studied in exquisite detail, the findings in *O. tipulae* allowed us to uncover a role for *mig-13* in *C. elegans* vulval development. The precise involvement of *mig-13* in vulva development remains an open question. MIG-13 in *C. elegans* was known to be required for the anterior migration of the QR neuroblast (Sym *et al.* 1999, p. 1). Lrp12, a mouse *trans*-membrane protein containing CUB and LDL repeats, is expressed in populations of migrating and polarized neurons during corticogenesis (Schneider *et al.* 2011) and can partially rescue a *C. elegans mig-13* mutant (Wang *et al.* 2013). Although the mechanism of action of *mig-13* in neuroblast migration remains elusive, it likely acts cell autonomously to polarize the actin skeleton at the leading edge of the migrating cell (Wang *et al.* 2013). As reported previously (Sym *et al.* 1999), we were unable to detect *mig-13* expression in the Pn.p vulva precursor cells in *C. elegans*, but this could be due to a weak expression [as in QR neurons (Wang *et al.* 2013)]. A cell-autonomous role of MIG-13 in migration of Pn.p cells could explain the centering phenotype observed in *O. tipulae*, although the mechanism of its effect on competence is less clear. In *C. elegans*, although vulval precursor cells move (Grimbert *et al.* 2016), this movement could be of reduced importance for proper centering of the cell fate pattern on P6.p. This reduced role would explain why *cov* phenotypes are not penetrant. It is tempting to speculate that MIG-13 acts as a receptor, but no ligand or protein partner has been identified. Finally, since a mixture of competence and centering phenotypes have been reported in Wnt mutants of *C. elegans* (Eisenmann *et al.* 1998; Milloz *et al.* 2008; Grimbert *et al.* 2016), it will be important to test whether the Wnt and the *mig-13* pathways interact during vulva development, and whether the Wnt pathway is disrupted in other *O. tipulae cov* mutants. Interestingly, *mig-13* appears to be absent from the *P. pacificus* genome. Comparing vulva development in the three species will provide a useful framework to polarize evolutionary changes and understand the genetic basis of phenotypic change and stasis despite pervasive developmental system drift.

In conclusion, we have shown how a draft *de novo* genome assembly can be used to identify phenotype-causing mutations in a nonmodel species. Our method does not require physical or genetic maps of the genome. In addition to further understanding of key developmental mechanisms in nematodes, the new assembly will be a useful resource for phylum-wide nematode genome analyses. We validated our approach by successfully identifying *Oti-mig-13* as the gene responsible for the vulva mutant phenotypes described in *cov-3* mutants (Louvet-Vallée *et al.* 2003). Our results pave the way for the identification of further *O. tipulae* mutations. Mapping-by-sequencing provides further linkage information, creating a virtuous circle between genome assembly and mutant mapping. More broadly, this work shows that the combination of better assembly techniques and mapping-by-sequencing now makes forward genetics realistic in nonmodel species.

## Supplementary Material

Supplemental material is available online at www.genetics.org/lookup/suppl/doi:10.1534/genetics.117.203521/-/DC1.
