# Supplementary figures and images for: Toward Universal Forward Genetics: Using a Draft Genome Sequence of the Nematode Oscheius tipulae To Identify Mutations Affecting Vulva Development
Source: Genetics. 2017 Jun 19;206(4):1747–61. doi: 10.1534/genetics.117.203521 (PMC5560785; doi:10.1534/genetics.117.203521)

A

Paired-End Insert size

Error Inserts

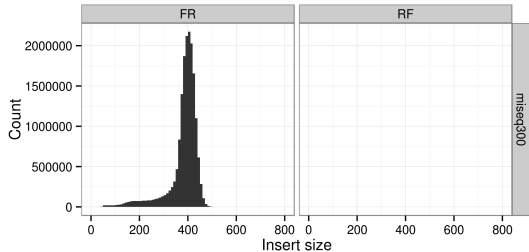

B

Error Inserts

Mate-pair Insert size

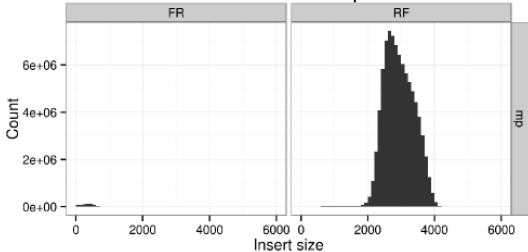

Supplement: Supplementary file 2 [file 1747FigureS1.pdf]

A

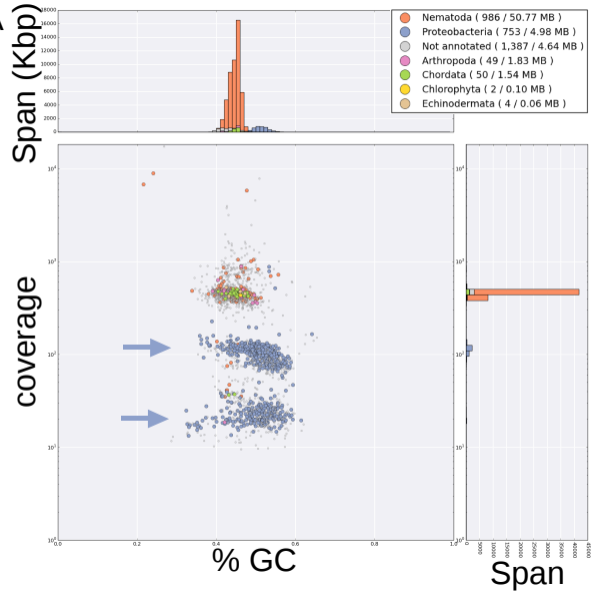

B

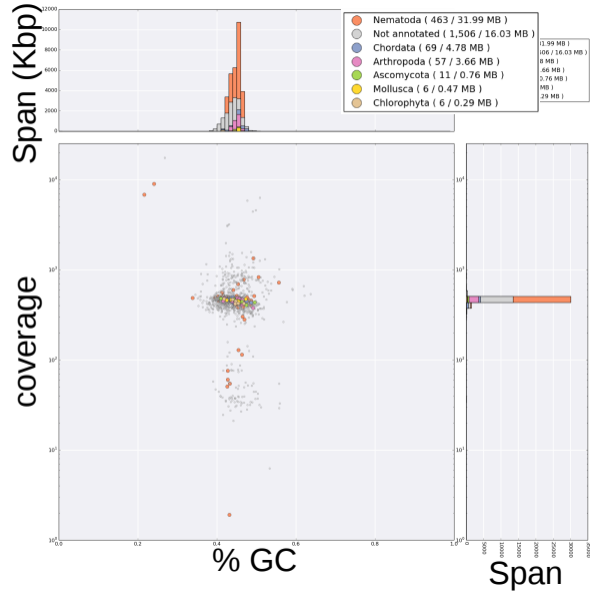

Supplement: Supplementary file 3 [file 1747FigureS2.pdf]

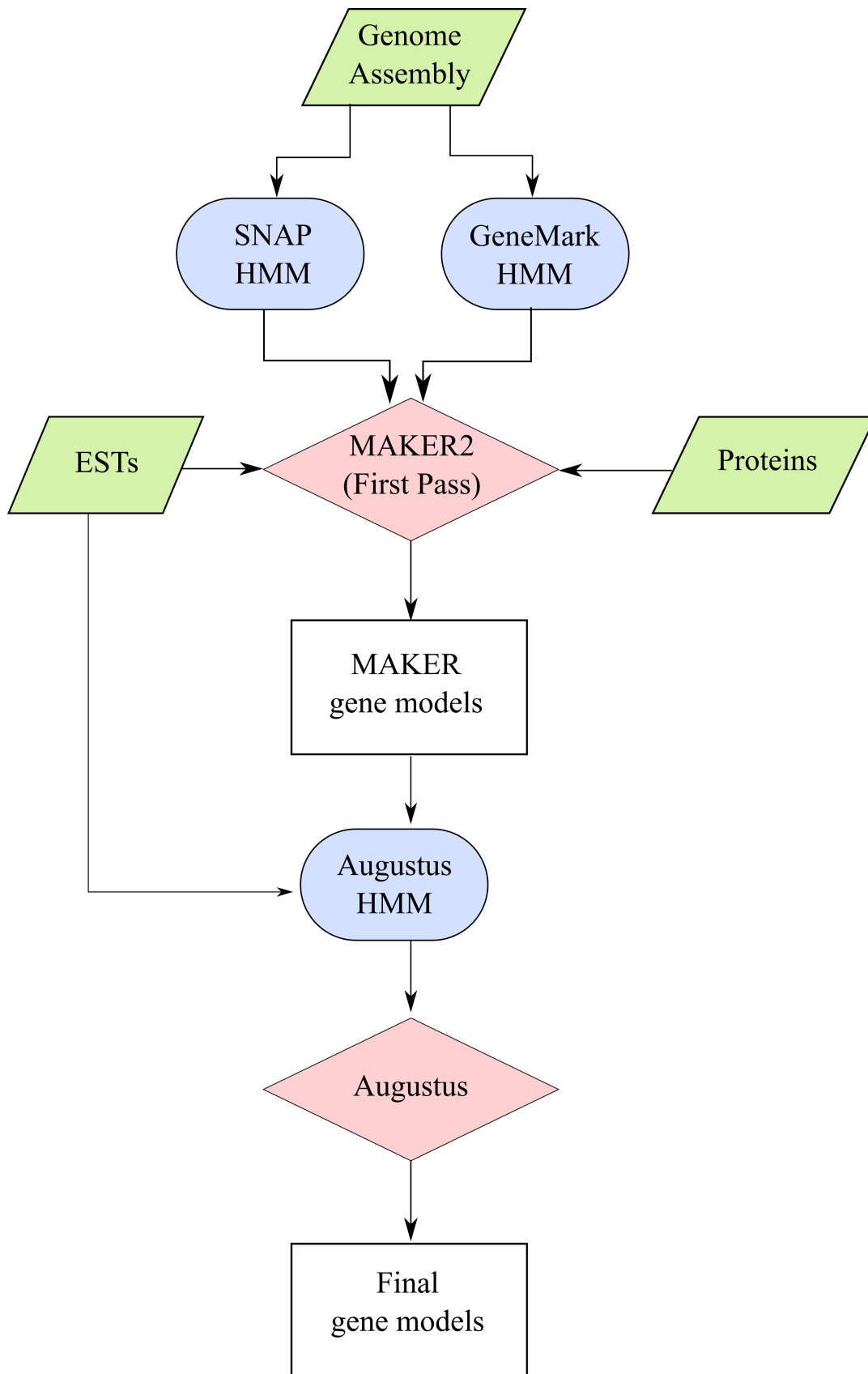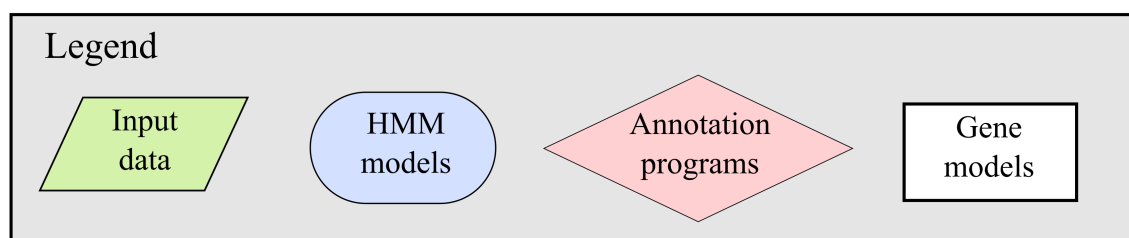

Supplement: Supplementary file 4 [file 1747FigureS3.pdf]

# Figure S4

A

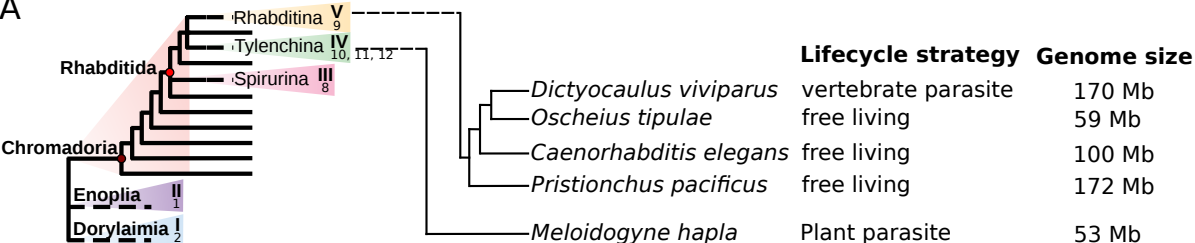

B

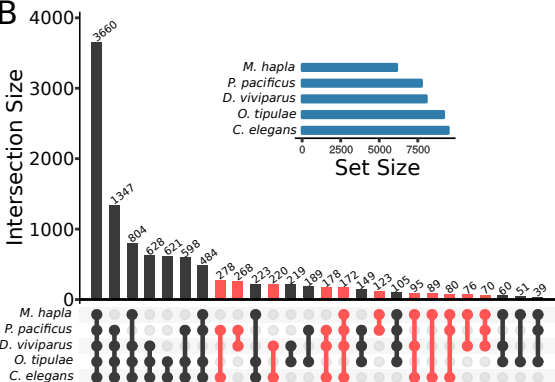

C

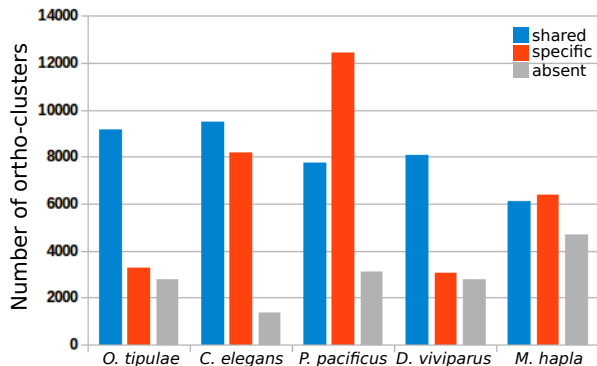

Supplement: Supplementary file 5 [file 1747FigureS4.pdf]

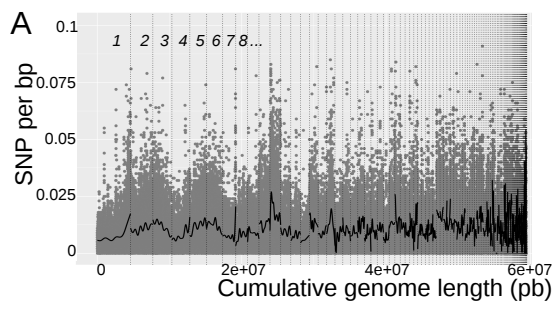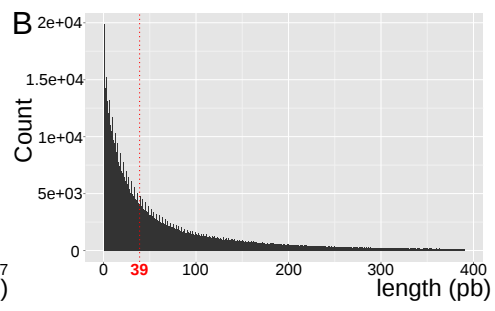

Supplement: Supplementary file 6 [file 1747FigureS5.pdf]

# A scaffold 1

coverage

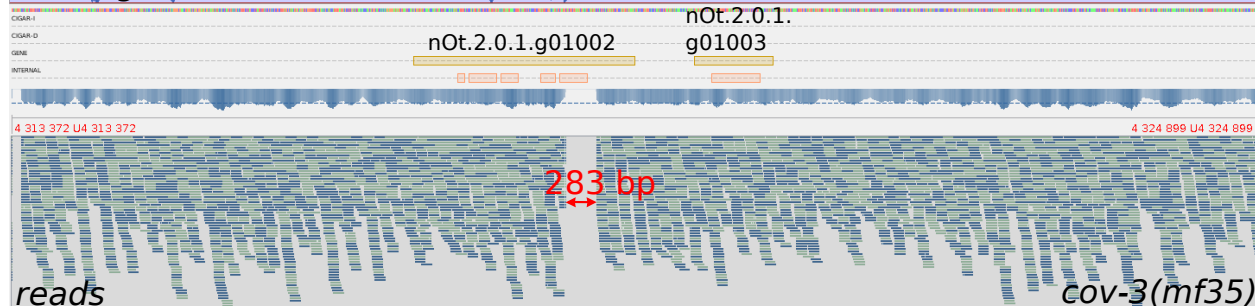

B

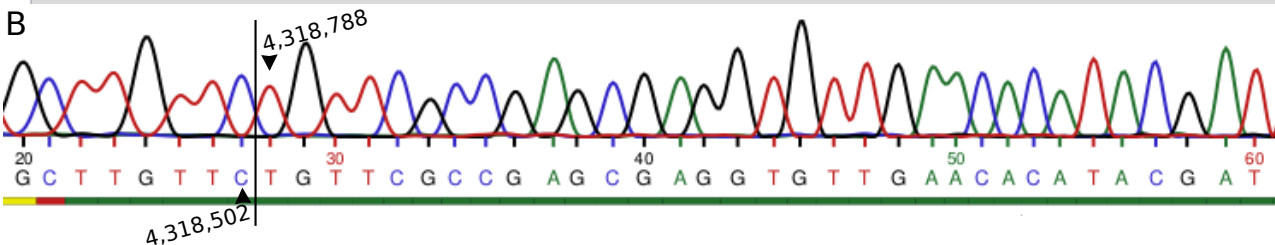

# C scaffold 1

coverage

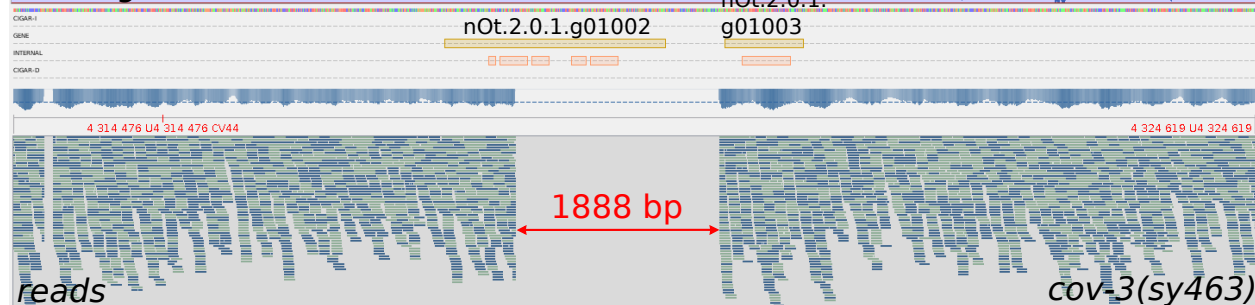

D

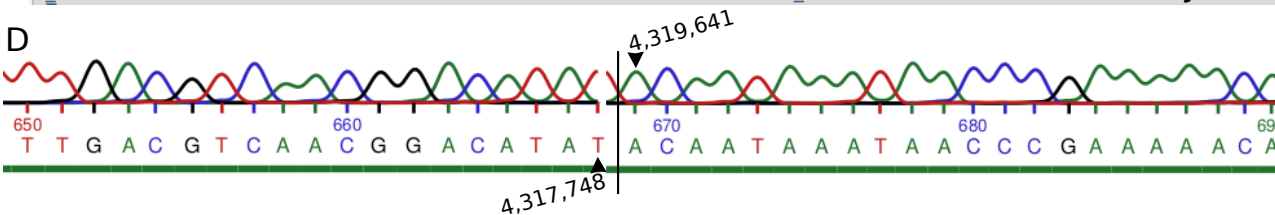

Supplement: Supplementary file 7 [file 1747FigureS6.pdf]

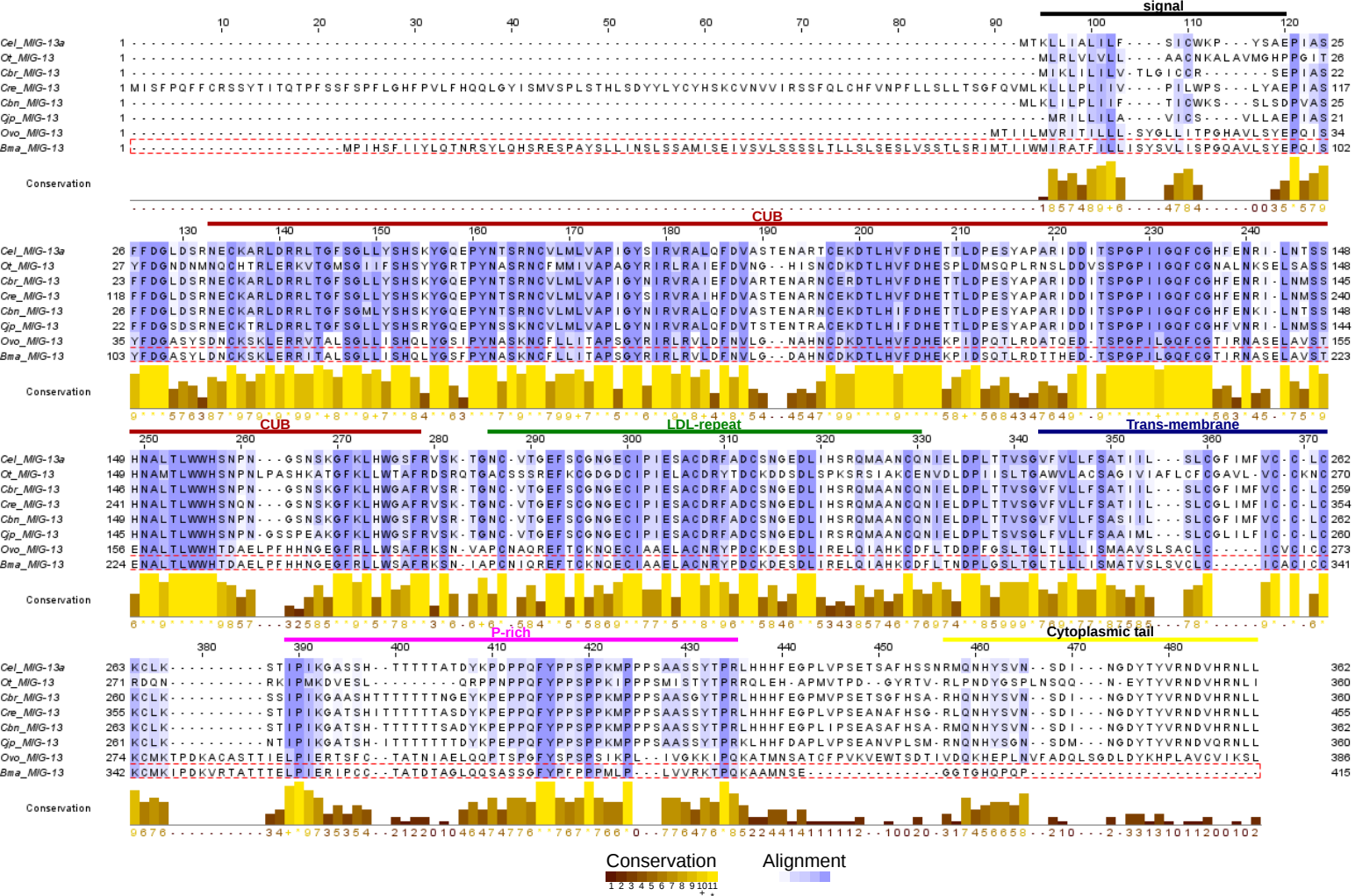

Supplement: Supplementary file 8 [file 1747FigureS7.pdf]

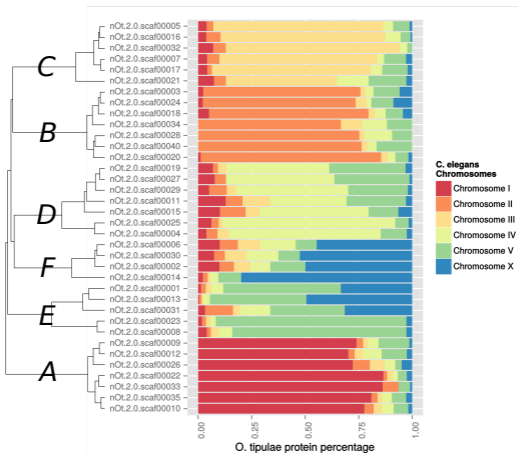

Supplement: Supplementary file 10 [file 1747FigureS9.pdf]

A

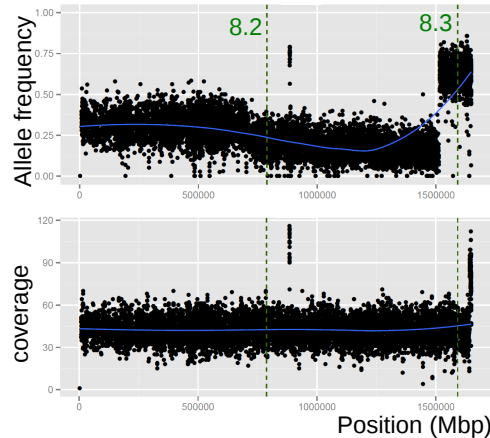

B

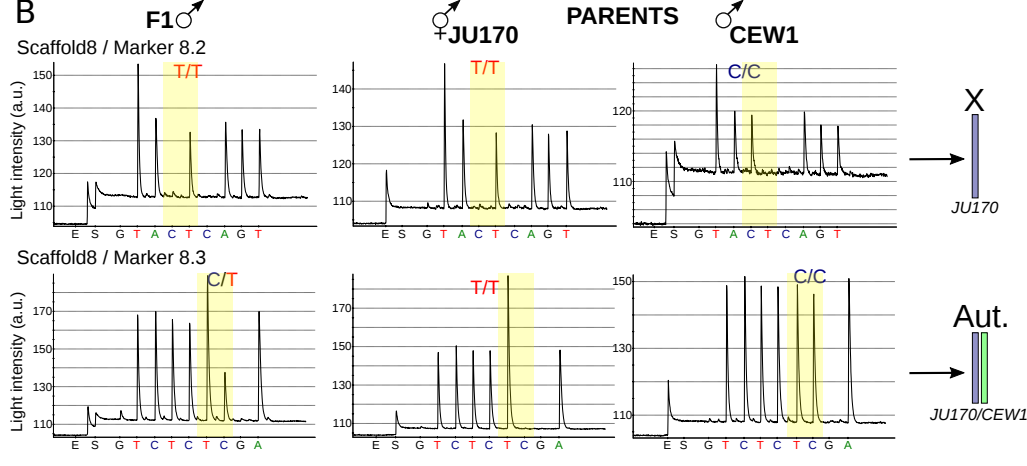

Supplement: Supplementary file 11 [file 1747FigureS10.pdf]
