# Supplementary material for: Toward Universal Forward Genetics: Using a Draft Genome Sequence of the Nematode Oscheius tipulae To Identify Mutations Affecting Vulva Development
Source: Genetics. 2017 Jun 19;206(4):1747–61. doi: 10.1534/genetics.117.203521 (PMC5560785; doi:10.1534/genetics.117.203521)
Supplement: Supplementary file 12 [file 1747TableS1.pdf]

**Table S1 : Genomic sequencing data used in this study for draft assembly**

| Library | Run | Type | Year | Instrument | Insert size (bp) | Read Length (bp) | No. Reads | No. of bases | After Trimming and Error correction | | | Used for Assembly | Final coverage |
|---|---|---|---|---|---|---|---|---|---|---|---|---|---|
| | | | | | | | | | No. Reads | No. of bases | % (vs raw reads) | | |
| 2009_PE-Lib1 | 090828_6_1 & 6_2 | PE | 2009 | Solexa | 250 | 51 | 5,581,821 | 569,345,742 | 4,503,739 | 435,583,880 | 76.50 | No | ~7 |
| 2010_PE-Lib1 | 100325_7_1 & 7_2 | PE | 2010 | Solexa | 250 | 101 | 7,757,564 | 1,567,027,928 | 7,091,729 | 1,305,387,838 | 83.30 | No | ~22 |
| 2010_PE-Lib2 | 100226_7 | SE | 2010 | Solexa | -- | 101 | 7,415,844 | 749,000,244 | 6,859,260 | 572,200,184 | 76.39 | No | ~10 |
| 2014-PE-Lib | 131101[...]_1 & _2 | PE | 2014 | MiSeq | 400 | 100 | 6,534,850 | 1,306,970,000 | 5,813,228 | 1,145,842,522 | 87.67 | Yes | ~19 |
| 2014-PE-Lib | 140213[ ...]_1 & _2 | PE | 2014 | MiSeq | 400 | 300 | 20,703,528 | 12,422,116,800 | 18,495,175 | 8,931,821,538 | 71.90 | Yes | ~150 |
| 2014-MP-Lib | 140225[...]_1 & _2 | MP | 2014 | HiSeq | 3000 | 100 | 145,536,412 | 29,107,282,400 | 100,981,836 | 18,121,587,583 | 62.25 | Yes | ~300 |
