# Supplementary material for: Toward Universal Forward Genetics: Using a Draft Genome Sequence of the Nematode Oscheius tipulae To Identify Mutations Affecting Vulva Development
Source: Genetics. 2017 Jun 19;206(4):1747–61. doi: 10.1534/genetics.117.203521 (PMC5560785; doi:10.1534/genetics.117.203521)
Supplement: Supplementary file 13 [file 1747TableS2.pdf]

Table S2: **Comparisons of genomic characteristics between different nematode species[1].**

		Oscheius tipulae	***Caenorhabditis elegans***	***Pristionchus pacificus***	***Dictyocaulus viviparus***	***Meloidogyne hapla***
Gene-related content	Genome size (Mb)	59	100	172	170	53
	Total intergenic span (Mb) (% of total genome)	21 (35.5%)	33 (33.0%)	78 (45.3%)	85 (50.0%)	27 (50.9%)
	Total genic span (Mb) (% of total genome)	38 (64.5%)	63 (63.0%)	94 (54.7%)	85 (50.0%)	26 (49.1%)
	Number of genes	14 938	20 240	24 216	14 306	14 420
	Number of genes / Mbp	251	202	141	84	272
	Mean / median transcript length (bp)	1368 / 1032	1385 / 1087	1005 / 732	1806 / 1471	1048 / 753
	Mean / median exon length (bp)	160 / 132	225 / 162	97 / 85	192 / 123	171 / 144
	Mean / median number of exons per gene	8.5 / 7	6 / 5	10 / 8	9 / 7	6 / 4
	Mean intron length (bp)	160	339	309	492	152
Repetitive content	SINEs (bp) (% of total genome)	0 (0.00 %)	133 625 (0.13 %)	313 002 (0.18 %)	57 714 (0.03 %)	0 (0.00 %)
	LINEs (bp) (% of total genome)	322 641 (0.54 %)	549 204 (0.55 %)	2 955 740 (1.71 %)	12 106 172 (7.15 %)	321 343 (0.61 %)
	LTR elements (bp) (% of total genome)	31 766 (0.05 %)	466 719 (0.47 %)	1 558 647 (0.90 %)	2 392 810 (1.41 %)	381 433 (0.72 %)
	DNA elements (bp) (% of total genome)	591 785 (1.00 %)	9 916 881 (9.89 %)	4 049 024 (2.35 %)	1 787 766 (1.06 %)	418 522 (0.79 %)
	Small RNA (bp) (% of total genome)	31 460 (0.05 %)	54 858 (0.05 %)	1 101 189 (0.64 %)	114 309 (0.07 %)	60 265 (0.11 %)
	Satellites (bp) (% of total genome)	53 (0.00 %)	804 613 (0.80 %)	162 136 (0.09 %)	103 (0.00 %)	8 885 (0.02 %)
	Simple repeats (bp) (% of total genome)	474 999 (0.80 %)	1 225 772 (1.22 %)	2 133 388 (1.24 %)	1 700 554 (1.00 %)	1 408 980 (2.66 %)
	Low complexity repeats (bp) (% of total genome)	77 021 (0.13 %)	282 807 (0.28 %)	628 600 (0.36 %)	303 457 (0.18 %)	726 847 (1.37 %)
	Unclassified repeats (bp) (% of total genome)	3 4746 07 (5.84 %)	5 505 020 (5.49 %)	19 555 176 (11.34 %)	18 332 001 (10.82 %)	2 006 484 (3.78 %)
	Total (bp) (% of total genome)	4 996 942 (8.40 %)	18 722 493 (18.67 %)	32 372 708 (18.77 %)	36 754 436 (21.70 %)	5 329 669 (10.05 %)
	Repeats in genic region (bp) (% of repeat bp)	2225243 (44.53%)	11477253 (61.31%)	16198756 (50.04%)	15735469 (42.81%)	1824665 (34.24%)
	Repeats in intergenic region (bp) (% of repeat bp)	2771699 (55.47%)	7245240 (38.69%)	16173952 (49.96%)	21018967 (57.19%)	3505004 (65.76%)

(1) Phylogenetic relationships between these species are indicated in Figure S4.
