# Supplementary material for: Toward Universal Forward Genetics: Using a Draft Genome Sequence of the Nematode Oscheius tipulae To Identify Mutations Affecting Vulva Development
Source: Genetics. 2017 Jun 19;206(4):1747–61. doi: 10.1534/genetics.117.203521 (PMC5560785; doi:10.1534/genetics.117.203521)
Supplement: Supplementary file 14 [file 1747TableS3.pdf]

<u>Table S3</u>: Mutant strains of *Oscheius tipulae* used in this study

**Allele**	**Locus**	**Mutagen**	**Strain**	**Used for**	**Reference**	**Sequencing data**
mf35	*cov-3*	EMS	JU54	Mapping-by-sequencing, gene identification, validation by Sanger sequencing and linkage group building	(Louvet-Vallée *et al.* 2003)	PRJEB19952
sy463	*cov-3*	TMP-UV	PS2456		(Louvet-Vallée *et al.* 2003)	PRJEB19960
mf79	*cov-3*	TMP-UV	JU111	Validation by Sanger sequencing	(Louvet-Vallée *et al.* 2003)	Not applicable
mf80	*cov-3*	EMS	JU113	Validation by Sanger sequencing	(Louvet-Vallée *et al.* 2003)	Not applicable
mf53	*cov-1*	EMS	JU71	Mapping-by-sequencing and Linkage group building	(Louvet-Vallée *et al.* 2003)	PRJEB19955
mf57	*cov-2*	EMS	JU85	Mapping-by-sequencing and Linkage group building	(Louvet-Vallée *et al.* 2003 p.)	PRJEB19956
sy465	*cov-4*	EMS	PS2458	Mapping-by-sequencing and Linkage group building	(Louvet-Vallée *et al.* 2003)	PRJEB19961
sy493	*cov-4*	EMS	PS2565	Mapping-by-sequencing and Linkage group building	(Louvet-Vallée *et al.* 2003)	PRJEB19962
mf34	*cov-5*	EMS	JU74	Mapping-by-sequencing and Linkage group building	(Louvet-Vallée *et al.* 2003)	PRJEB19951
sy460	*cov-6*	EMS	JU102	Mapping-by-sequencing and Linkage group building	(Louvet-Vallée *et al.* 2003)	PRJEB19925
sy466	*cov-7*	EMS	JU105	Mapping-by-sequencing and Linkage group building	(Louvet-Vallée *et al.* 2003 p.)	PRJEB19924
mf54	*cov-8*	EMS	JU78	Mapping-by-sequencing and Linkage group building	(Louvet-Vallée *et al.* 2003 p.)	PRJEB19923
mf89	*cov-9*	TMP-UV	JU172	Mapping-by-sequencing and Linkage group building	(Louvet-Vallée *et al.* 2003 p.)	PRJEB19922
mf86	*iov-1*	TMP-UV	JU163	Mapping-by-sequencing and Linkage group building	(Dichtel-Danjoy and Félix 2004)	PRJEB19957
mf76	*iov-2*	TMP-UV	JU100	Mapping-by-sequencing and Linkage group building	(Dichtel-Danjoy and Félix 2004)	PRJEB19921
sy447	*iov-3*	EMS	PS2373	Mapping-by-sequencing and Linkage group building	(Dichtel-Danjoy and Félix 2004)	PRJEB19958
mf52	*iov-3*	EMS	JU69	Mapping-by-sequencing and Linkage group	(Dichtel-	PRJEB19954

Allele	Locus	Mutagen	Strain	Used for	Reference	Sequencing data
				building	Danjoy and Félix 2004)	
mf67	*iov-4*	EMS	JU101	Mapping-by-sequencing and Linkage group building	(Dichtel-Danjoy and Félix 2004)	PRJEB19919
mf99	*iov-5*	EMS	JU386	Mapping-by-sequencing and Linkage group building	(Dichtel-Danjoy and Félix 2004)	PRJEB19917
mf33	*unc*	EMS	JU46	Mapping-by-sequencing and Linkage group building	(Félix *et al.* 2000); this study	PRJEB19913
