# Supplementary material for: Toward Universal Forward Genetics: Using a Draft Genome Sequence of the Nematode Oscheius tipulae To Identify Mutations Affecting Vulva Development
Source: Genetics. 2017 Jun 19;206(4):1747–61. doi: 10.1534/genetics.117.203521 (PMC5560785; doi:10.1534/genetics.117.203521)
Supplement: Supplementary file 15 [file 1747TableS4.pdf]

Table S4: Software and command lines/main options

Tool Name	Reference	Version	Relevant Parameters
Cutadapt	DOI:10.14806/ej.17.1.200	v1.6	-O 5
Trimmomatic	https://doi.org/10.1093/bioinformatics/btu170	0,32	LEADING:3 TRAILING:3 SLIDINGWINDOW:4:20 MINLEN:31
Quake	DOI: 10.1186/gb-2010-11-11-r116	0,3	-k 19
CLC bio	http://www.clcbio.com/products/clc-genomics-workbench/	4.2.1	
blobtools	https://doi.org/10.3389/fgene.2013.00237<br>http://doi.org/10.5281/zenodo.177699		
bowtie2	DOI: 10.1186/gb-2009-10-3-r25	2.2.0	
samtools	https://doi.org/10.1093/bioinformatics/btp352	1.1 or later	
ncbi-blast+	DOI: 10.1186/1471-2105-10-421	2.2.30	
sga	doi: 10.1101/gr.126953.111	v.0.10.13	
kmergenie	https://doi.org/10.1093/bioinformatics/btt310	1,6741	-l 25 -s 2
preqc	arXiv:1307.8026		
ABySS	doi: 10.1101/gr.089532.108	1.5.2	k=69
Velvet	doi: 10.1101/gr.074492.107	1.2.10	kmer size 69
SPAdes	doi:10.1089/cmb.2012.0021	3.1.1	--only-assembler --careful
SOAPdenovo	DOI: 10.1186/2047-217X-1-18	v. r240	pair_num_cutoff=5 map_len=35 -K=69 -R
ALLPATHS-LG	doi: 10.1073/pnas.1017351108	v. 50960	
MaSuRCA	https://doi.org/10.1093/bioinformatics/btt476	2.3.2	
Ray	doi:10.1089/cmb.2009.0238	2.3.1	-k 69
REAPR	DOI: 10.1186/gb-2013-14-5-r47	1.0.17	
ALE	doi: 10.1093/bioinformatics/bts723	20130717	
CEGMA	https://doi.org/10.1093/bioinformatics/btm071	2,4	
RepeatModeler	http://www.repeatmasker.org/	1.0.8	
RepeatMasker	http://www.repeatmasker.org/	4.0.3	
SNAP	doi: 10.1186/1471-2105-5-59	2013-11-29	
GeneMark-ES	doi: 10.1002/0471250953.bi0406s35	2,3	--BP OFF -max_nnn 500
MAKER2	DOI: 10.1186/1471-2105-12-491	2,31	
Augustus	doi: 10.1093/bioinformatics/btr010	3.2.1	autoAug.pl with 3 rounds of training
bwa	https://doi.org/10.1093/bioinformatics/btp324	0.7.12-r1044 or later	mem -aM
Picard	http://broadinstitute.github.io/picard/	1.110 or later	Tools: AddOrReplaceReadGroups + MarkDuplicates + BuildBamIndex
GATK	DOI: 10.1002/0471250953.bi1110s43	3.3-0 or later	Tools: RealignerTargetCreator + IndelRealigner +BaseRecalibrator<br>+AnalyzeCovariates+PrintReads+ HaplotypeCaller
FastQC	http://www.bioinformatics.babraham.ac.uk/projects/fastqc/	0.11.1	--nogroup
Qualimap	doi: 10.1093/bioinformatics/bts503	v2.0	
snpEff	http://dx.doi.org/10.4161/fly.19695	4.1g or later	
Tablet	https://doi.org/10.1093/bib/bbs012	1.14.04.10 or later	
Jellyfish	doi:10.1093/bioinformatics/btr011	1.1.11	
Orthofinder	DOI: 10.1186/s13059-015-0721-2	1.1.4	-I 3
