# Supplementary material for: Toward Universal Forward Genetics: Using a Draft Genome Sequence of the Nematode Oscheius tipulae To Identify Mutations Affecting Vulva Development
Source: Genetics. 2017 Jun 19;206(4):1747–61. doi: 10.1534/genetics.117.203521 (PMC5560785; doi:10.1534/genetics.117.203521)
Supplement: Supplementary file 16 [file 1747TableS5.pdf]

Table S5 : **Statistics of preliminary assembly using different set of sequencing data**

	Old sequencing data	New sequencing data	All sequencing data together
Span	55,671,395	64,014,276	63,852,055
No. of contigs	18,266	3,243	3,436
Longest contig	47,141	987,545	922,06
N50	5,71	71,403	70,491
No. N's	6,831	6,819	8,065
GC %	44.9	45	45
