# Supplementary material for: Toward Universal Forward Genetics: Using a Draft Genome Sequence of the Nematode Oscheius tipulae To Identify Mutations Affecting Vulva Development
Source: Genetics. 2017 Jun 19;206(4):1747–61. doi: 10.1534/genetics.117.203521 (PMC5560785; doi:10.1534/genetics.117.203521)
Supplement: Supplementary file 17 [file 1747TableS6.pdf]

Table S6: List of primers used in this study

Purpose	Oligoname	sequence[a]	scaffold	primer starts at / SNP position[b]	Polymorphism (CEW1 → JU170)[c]
Sanger-sequencing	Ot-mig13_F	TTTGTGAGCACTCGGCCATC	1	41 bp upstream of ATG	
Sanger-sequencing	Ot-mig13_R	TCGAATGTATTCCATGTACACTG	1	541 bp downstream of STOP	
Sanger-sequencing	Ot-mig13_InternF	ATGTCGACCTTGATCCGATC	1	Start of exon6	
Sanger-sequencing	Ot-mig13_InternF2	GGTATAATCTTCTCACACAGC	1	Start 523 bp downstream ATG (unspliced)	
Pyrosequencing	1.1_F	GGCAAAATTATCCACGAATTAAA	1	2227845	T -> G
Pyrosequencing	1.1_R	TAGCAGGATACGACTATCTTCAGAATCTGTTTTGGGACTCAT	1		
Pyrosequencing	1.1_S	TTACATAGCATCCAAATAAA	1		
Pyrosequencing	2.2 F	GTGACGTACTAGCAACGGCCCAGTCTGCACAGTAGAAG	2	1512781	A -> G
Pyrosequencing	2.2 R	TCCAGTGTCCACGTCTGTTTC	2		
Pyrosequencing	2.2 S	CGTCTGTTTCCATGAA	2		
Pyrosequencing	2.3_F	GTGACGTACTAGCAACGGGGTACATCTACTTGACGAGGATC	2	1542188	T -> C
Pyrosequencing	2.3_R	AAGAGCCACCTCCTAAAGAGAAA	2		
Pyrosequencing	2.3_S	AAGAGAAAGAGCACAAAAT	2		
Pyrosequencing	6.2 F	GTGACGTACTAGCAACGTAGGTTCGCAGCGCAGTT	6	1053740	A -> C
Pyrosequencing	6.2 R	ACGAGCCACTGAATTTGACA	6		
Pyrosequencing	6.2 S	CCCTCAGAGGCCCAGC	6		
Pyrosequencing	6.3_F	CAAGTTGCCGATCAATGGGA	6	2118798	G -> A
Pyrosequencing	6.3_R	TAGCAGGATACGACTATCACCTTCCTCCTGGTAGGATTCAAT	6		
Pyrosequencing	6.3_S	GAATCGGGCCTTTAC	6		
Pyrosequencing	8.2-F	TCCCAGCGAAAAAACGTCT	8	787997	C -> T
Pyrosequencing	8.2-R	TAGCAGGATACGACTATCGAAAGGAGCTAAACACGAGAGATT	8		
Pyrosequencing	8.2-S	CAGCGAAAAAACGTCT	8		
Pyrosequencing	8.3_F	GTGACGTACTAGCAACGGGCGGAAAATTTCAAACG	8	1591472	G -> A
Pyrosequencing	8.3_R	CCCCTTTTACAATGGCAAGTAG	8		
Pyrosequencing	8.3_S	TGACCGCTTTATATATCTG	8		
Pyrosequencing	13.2 F	GTGACGTACTAGCAACGCTCCCTGACTCTATCAGCACTCAC	13	668272	A -> G
Pyrosequencing	13.2 R	ACAAGGAAAAGGCCCGTCTCT	13		
Pyrosequencing	13.2 S	CCGTCTCTGTCTGCC	13		
Pyrosequencing	14.2 F	CAGTGAGAACAAACCAGGTCTGC	14	619189	C -> A
Pyrosequencing	14.2 R	TAGCAGGATACGACTATCTGCTCATCAACTCTCGGGATCT	14		
Pyrosequencing	14.2 S	GCAAGGGTGGTCCAA	14		
Pyrosequencing	23.3_F	GTGACGTACTAGCAACGTCCGGTGAATGGATACAAACTA	23	440000	T -> A
Pyrosequencing	23.3_R	CGACTCTTACCAGCGAAGAAAT	23		
Pyrosequencing	23.3_S	TGACATGATTAGAGATTGTT	23		
Pyrosequencing	30.3_F	AAGTGTCGAGGAGCTGTTGG	30	300627	T -> A
Pyrosequencing	30.3_R	TAGCAGGATACGACTATCGAAGGGTATTGAGCAGGTTCTG	30		
Pyrosequencing	30.3_S	ATTGCTGTGGACGTG	30		
Pyrosequencing	31.1_F	TCCCTCGGCTGTGTATTCA	31	257058	TAA ->T
Pyrosequencing	31.1_R	TAGCAGGATACGACTATCGAATCCGCTCCAGCAATG	31		
Pyrosequencing	31.1_S	AACGAACAAATACATGAAA	31		

(a) Green and blue sequences are Universal Sequence Forward and Reverse, respectively. They were added as queue to the sequences matching the *Oscheius* genome.
(b) SNP position in the corresponding scaffold, in bp, in the assembly nOt.2.0.
(c) in the forward strain of the assembly nOt.2.0.
