# Supplementary material for: Toward Universal Forward Genetics: Using a Draft Genome Sequence of the Nematode Oscheius tipulae To Identify Mutations Affecting Vulva Development
Source: Genetics. 2017 Jun 19;206(4):1747–61. doi: 10.1534/genetics.117.203521 (PMC5560785; doi:10.1534/genetics.117.203521)
Supplement: Supplementary file 9 [file 1747FigureS8.pdf]

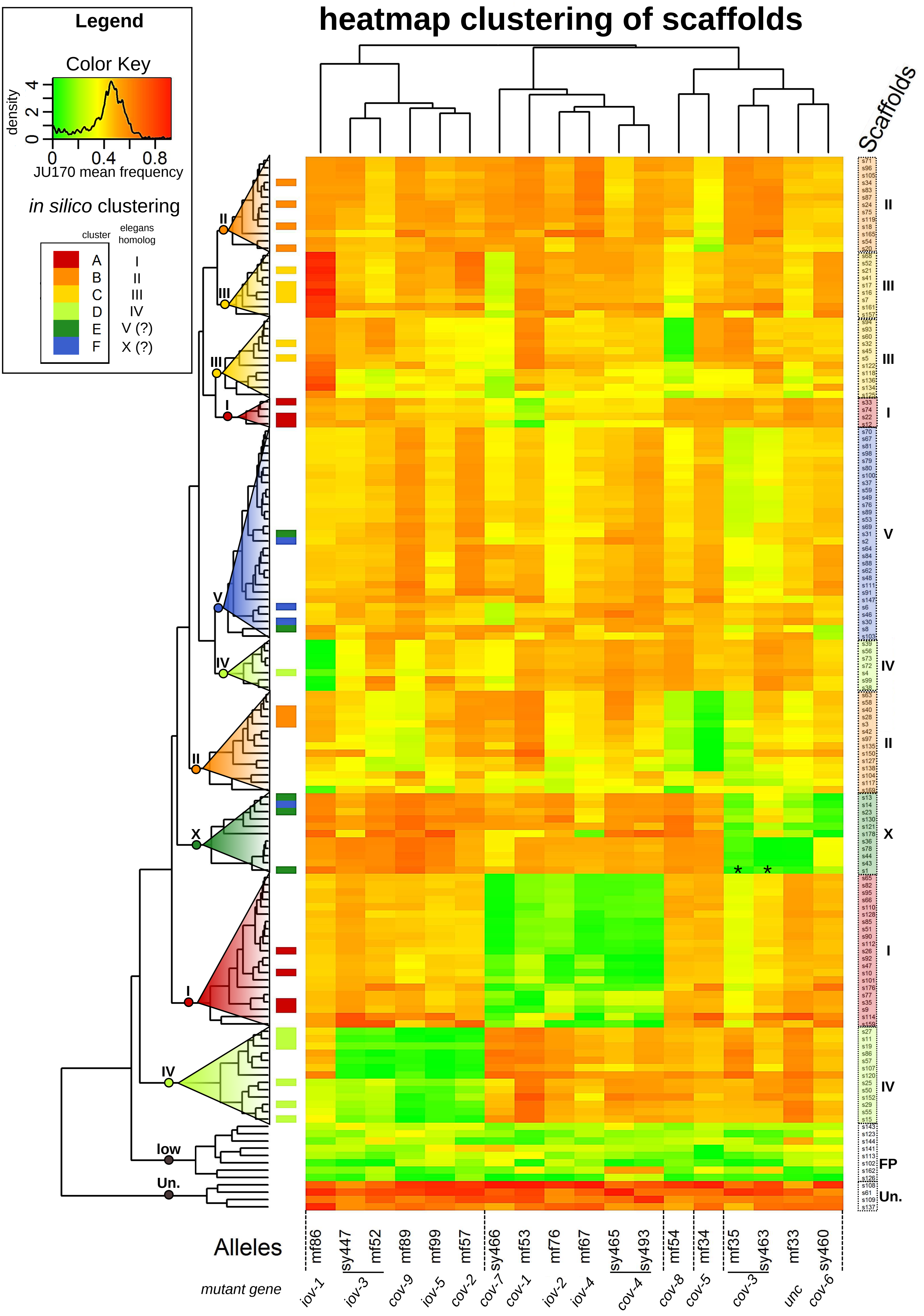
heatmap clustering of scaffolds
Legend
Color Key
density
0
2
4
0
0.4
0.8
JU170 mean frequency
in silico clustering
cluster
elegans homolog
A I
B II
C III
D IV
E V (?)
F X (?)
Scaffolds
II
III
III
I
V
IV
II
X
I
IV
FP
Un.
low
Un.
Alleles
mf86
sy447
mf52
mf89
mf99
mf57
sy466
mf53
mf76
mf67
sy465
sy493
mf54
mf34
mf35
sy463
mf33
sy460
mutant gene
iov-1
iov-3
cov-9
iov-5
cov-2
cov-7
cov-1
iov-2
iov-4
cov-4
cov-8
cov-5
cov-3
unc
cov-6
